# Genetically mimicked effects of ASGR1 inhibitors on all-cause mortality and health outcomes: a drug-target Mendelian randomization study and a phenome-wide association study

**DOI:** 10.1186/s12916-023-02903-w

**Published:** 2023-07-03

**Authors:** Guoyi Yang, C. Mary Schooling

**Affiliations:** 1 School of Public Health, Li Ka Shing Faculty of Medicine, The University of Hong Kong, Hong Kong, China; 2 Graduate School of Public Health and Health Policy, City University of New York, New York, USA

**Keywords:** All-cause mortality, ASGR1 inhibitors, Mendelian randomization, Phenome-wide association study, Cardiovascular disease

## Abstract

**Background:**

Asialoglycoprotein receptor 1 (ASGR1) is emerging as a potential drug target to reduce low-density lipoprotein (LDL)-cholesterol and coronary artery disease (CAD) risk. Here, we investigated genetically mimicked ASGR1 inhibitors on all-cause mortality and any possible adverse effects.

**Methods:**

We conducted a drug-target Mendelian randomization study to assess genetically mimicked effects of ASGR1 inhibitors on all-cause mortality and 25 *a priori* outcomes relevant to lipid traits, CAD, and possible adverse effects, i.e. liver function, cholelithiasis, adiposity and type 2 diabetes. We also performed a phenome-wide association study of 1951 health-related phenotypes to identify any novel effects. Associations found were compared with those for currently used lipid modifiers, assessed using colocalization, and replicated where possible.

**Results:**

Genetically mimicked ASGR1 inhibitors were associated with a longer lifespan (3.31 years per standard deviation reduction in LDL-cholesterol, 95% confidence interval 1.01 to 5.62). Genetically mimicked ASGR1 inhibitors were inversely associated with apolipoprotein B (apoB), triglycerides (TG) and CAD risk. Genetically mimicked ASGR1 inhibitors were positively associated with alkaline phosphatase, gamma glutamyltransferase, erythrocyte traits, insulin-like growth factor 1 (IGF-1) and C-reactive protein (CRP), but were inversely associated with albumin and calcium. Genetically mimicked ASGR1 inhibitors were not associated with cholelithiasis, adiposity or type 2 diabetes. Associations with apoB and TG were stronger for ASGR1 inhibitors compared with currently used lipid modifiers, and most non-lipid effects were specific to ASGR1 inhibitors. The probabilities for colocalization were > 0.80 for most of these associations, but were 0.42 for lifespan and 0.30 for CAD. These associations were replicated using alternative genetic instruments and other publicly available genetic summary statistics.

**Conclusions:**

Genetically mimicked ASGR1 inhibitors reduced all-cause mortality. Beyond lipid-lowering, genetically mimicked ASGR1 inhibitors increased liver enzymes, erythrocyte traits, IGF-1 and CRP, but decreased albumin and calcium.

**Supplementary Information:**

The online version contains supplementary material available at 10.1186/s12916-023-02903-w.

## Background

Asialoglycoprotein receptor 1 (ASGR1) is the major subunit of asialoglycoprotein receptor (ASGPR), a liver-specific lectin that plays a role in the homeostasis of glycoprotein [1]. Variants in *ASGR1* are associated with lower non-high-density lipoprotein (non-HDL)-cholesterol and a lower risk of coronary artery disease (CAD) [2, 3]. Anti-ASGR1 neutralizing antibodies in mice show synergistic effects on serum cholesterol relative to some currently used lipid modifiers (i.e. statins and ezetimibe) [4], highlighting ASGR1 as a possible therapeutic target for lowering cholesterol and preventing cardiovascular diseases (CVD) [5].

Beyond lipid modification, the consequences of inhibiting ASGR1 are uncertain, raising the possibility of potentially important non-lipid effects. A loss-of-function variant in *ASGR1* (an intronic 12-base-pair deletion (del12)) confers a larger effect on CAD risk than is predicted by its effect on non-HDL-cholesterol in humans [2], suggesting non-lipid pathways also contribute to its athero-protective properties. Concerns have also been raised about the possibility of ASGR1 inhibitors having adverse effects on the liver or the biliary system [5]. ASGR1-deficient pigs have lower non-HDL-cholesterol but develop mild to moderate hepatic injury [6]. ASGR1 inhibitors may increase the risk of cholelithiasis through elevating biliary cholesterol excretion [4], similar to adenosine triphosphate-binding cassette transporters G5/8 (ABCG5/8) [7]. Furthermore, the overall effect of ASGR1 inhibitors on all-cause mortality remains unclear.

To address the gap, we performed a drug-target Mendelian randomization (MR) study [8], to assess genetically mimicked effects of ASGR1 inhibitors comprehensively in comparison with currently used lipid modifiers. First, we assessed genetically mimicked effects of ASGR1 inhibitors on all-cause mortality. Second, we investigated genetically mimicked effects of ASGR1 inhibitors on 25 traits selected *a priori* as known (lipid traits and CAD) [2, 3] or suspected (liver function and cholelithiasis) [5, 6] effects of inhibiting ASGR1, as well as adiposity and type 2 diabetes because these are well-known effects of statins [9] and have been suggested as general consequences of lowering LDL-cholesterol [10, 11]. Third, we conducted a phenome-wide association study (PheWAS), i.e. a wide-ranging genotype-to-phenotype scan [12], to examine the likely effects of ASGR1 inhibitors on a comprehensive range of health outcomes. Fourth, we conducted colocalization analysis to assess the plausibility of any associations found. We also replicated and assessed these associations by sex, where possible, because sex-specific effects are evident for some lipid modifiers [13, 14] and for LDL-cholesterol [15].

## Methods

### Study design

**Fig. 1:**
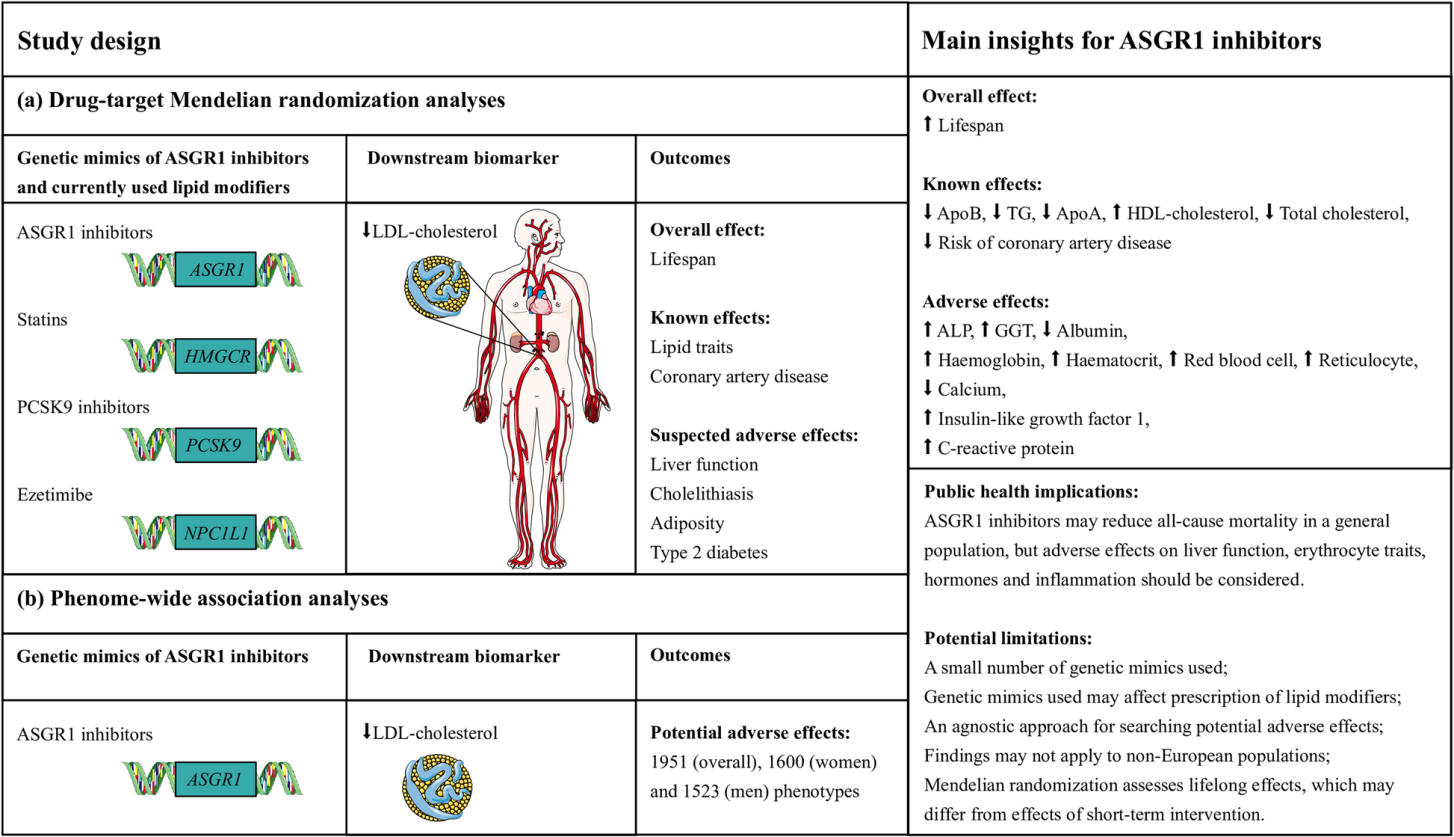
Study design and main insights for ASGR1 inhibitors. (a) ALP, alkaline phosphatase; ApoA, apolipoprotein A; ApoB, apolipoprotein B; GGT, gamma glutamyltransferase; HDL, high-density lipoprotein; LDL, low-density lipoprotein; TG, triglycerides

We used established SNPs selected from genes encoding the molecular targets of each therapy to mimic ASGR1 inhibitors [2] and currently used lipid modifiers (i.e. statins, proprotein convertase subtilisin/kexin type 9 (PCSK9) inhibitors and ezetimibe) [16]. First, we assessed associations with all-cause mortality in a meta-analysis of the UK Biobank and LifeGen [17]. Second, we assessed associations with 25 *a priori* outcomes relevant to lipid traits, CAD, liver function, cholelithiasis, adiposity and type 2 diabetes in the UK Biobank (http://www.nealelab.is/uk-biobank/). Third, we conducted a PheWAS of genetically mimicked ASGR1 inhibitors in the UK Biobank (http://www.nealelab.is/uk-biobank/). Fourth, we conducted colocalization analyses with LDL-cholesterol from Global Lipids Genetics Consortium (GLGC) and each of the significant outcomes in or near the *ASGR1* gene to examine whether any associations found were driven by a shared causal variant between exposure and outcome or were confounded by linkage disequilibrium [18]. Where possible, we used alternative genetic instruments and other publicly available summary statistics to replicate our findings. A summary of the study design is shown in Fig. 1, with more detail provided in Additional file 1: Supplemental Figure S1.

### UK Biobank

The UK Biobank recruited approximately 500,000 individuals (intended age 40–69 years, 45.6% men, 94% self-reported European ancestry) from 2006 to 2010 in England, Scotland and Wales [19]. Participants completed a variety of physical assessments, biological measurements and questionnaires including socioeconomic attributes, lifestyle and health-related conditions [19]. Follow-up information was obtained from record linkage to national medical and mortality records [19]. The genome-wide association study (GWAS) by Neale lab was restricted to people of white British ancestry (194,174 women and 167,020 men) to reduce confounding by population stratification and excluded individuals with excess relatedness or sex chromosome aneuploidy (http://www.nealelab.is/uk-biobank/). Summary statistics were adjusted for age, age^2^, inferred sex, age × inferred sex, age^2^ × inferred sex and the first 20 principal components in sex-combined analyses, and for age, age^2^ and the first 20 principal components in sex-specific analyses (http://www.nealelab.is/uk-biobank/).

### Genetic mimics of ASGR1 inhibitors and currently used lipid modifiers

A well-established genetic mimic of ASGR1 inhibitors was used, i.e. the A allele of rs186021206 (minor allele frequency (MAF) 0.46%), which proxies a loss-of-function variant in *ASGR1* (del12) (*r*^2^ = 0.86) and is associated with lower LDL-cholesterol and CAD risk [2]. Given rare genetic variants are not always included in GWAS and can have false-positive associations when linear regression is used for binary outcomes, we also identified more common genetic mimics of ASGR1 inhibitors. Specifically, we extracted independent (*r*^2^ < 0.001) common (MAF > 1%) variants in or near (± 1 Mb) *ASGR1* that were associated with LDL-cholesterol at genome-wide significance (*p* < 5 × 10^−8^).

We obtained genetic mimics of currently used lipid modifiers from published sources, which selected genetic variants from genes encoding the molecular targets of each therapy (6 SNPs from *HMGCR* for statins, 7 SNPs from *PCSK9* for PCSK9 inhibitors and 5 SNPs from *NPC1L1* for ezetimibe) [16]. Given all the SNPs for each lipid modifier were correlated (*r*^2^ ≥ 0.001), we only used the SNP most strongly associated with LDL-cholesterol in the main analysis and included all the relevant SNPs along with their correlation matrixes (Additional File 1: Supplemental Tables S1-3) in sensitivity analyses.

We expressed genetically mimicked effects of ASGR1 inhibitors and currently used lipid modifiers in effect sizes of LDL-cholesterol reduction (*N* = 842,660), taken from GLGC in people of European ancestry excluding the UK Biobank participants [20]. Pre-medication LDL-cholesterol for individuals on cholesterol-lowering medication was approximated by dividing LDL-cholesterol by 0.7 [20]. Summary statistics were adjusted for age, age^2^, sex, principal components of ancestry and study-specific covariates [20]. Given apolipoprotein B (apoB) has been suggested to account for the effects of LDL-cholesterol on CAD and lifespan [21, 22], we also expressed the estimates in effect sizes of apoB reduction (*N* = 342,590) obtained from a GWAS in the UK Biobank (http://www.nealelab.is/uk-biobank/).

### Genetic associations with all-cause mortality

We used parental attained age (i.e. current age or age at death) (*N* = 1,012,240) from a meta-analysis of the UK Biobank and LifeGen as a measure of all-cause mortality [17], because it reduces selection bias from inevitably only recruiting survivors and has more power than participant’s mortality status. Genetic associations with log protection ratio were adjusted for genotyping batch and array, the first 40 principal components of relatedness and participant sex for the UK Biobank [17], and for participant sex, the first 10 principal components and study-specific covariates for LifeGen [23]. Estimates were presented in terms of lifespan longer (positive) or shorter (negative) by multiplying the log protection ratio by 10 [17].

### Genetic associations with 25 *a priori* outcomes

We selected 25 health outcomes *a priori* based on relevance to known (lipid traits and CAD) or suspected (liver function, cholelithiasis, adiposity and type 2 diabetes) effects of ASGR1 inhibitors. Outcomes relevant to known effects were apoB, triglycerides (TG), lipoprotein(a) (Lp(a)), apolipoprotein A (apoA), HDL-cholesterol, total cholesterol, self-reported high cholesterol and CAD. Outcomes relevant to suspected effects were alkaline phosphatase (ALP), aspartate aminotransferase (AST), alanine transaminase (ALT), gamma glutamyltransferase (GGT), albumin, total bilirubin, direct bilirubin, cholelithiasis, body mass index (BMI), bodyweight, whole body fat mass, body fat percentage, waist circumference, hip circumference, glucose, glycated haemoglobin (HbA1c) and diagnosed diabetes. We obtained genetic associations with the 25 *a priori* outcomes from UK Biobank GWAS (http://www.nealelab.is/uk-biobank/).

### PheWAS phenotype selection

The UK Biobank GWAS provides 4541 different phenotypes in total (http://www.nealelab.is/uk-biobank/). We excluded duplicates, phenotypes selected *a priori*, phenotypes for external causes, socioeconomic factors, household attributes, employment, lifestyle (smoking, alcohol drinking, diet and physical activity), environmental attributes, family history, treatment/screening, other factors unlikely to reflect effects of ASGR1 inhibitors and phenotypes designated “None of the above”. We further excluded age at disease onset or diagnosis, age at death and also underlying causes of death because they exclude people without disease or remaining alive, which could generate selection bias. We excluded phenotypes where sex-combined or sex-specific summary statistics were not available, binary phenotypes with less than 100 cases and continuous or categorial ordered phenotypes with sample size less than 10,000 to ensure power as previously [13]. The flowchart of phenotype inclusion is shown in Additional file 1: Supplemental Figure S2.

Binary outcomes were classified according to International Classification of Disease (ICD)-10 chapters, i.e. infectious diseases, neoplasms, haematopoietic, endocrine, mental health, neurological, sense organs, circulatory, respiratory, digestive, dermatologic, musculoskeletal, genitourinary, obstetric, symptoms, injuries and poisonings and others. Continuous and categorial ordered phenotypes were grouped into physical measures, biomarkers and cognitive function as recommended by the UK Biobank (https://biobank.ndph.ox.ac.uk/showcase/cats.cgi). Genetic associations for binary outcomes obtained from linear regression were transformed into odds ratio (OR) using an established approximation [24].

### Colocalization analysis

We conducted colocalization analyses in a Bayesian framework to assess the posterior probability of a shared variant in or near (± 100 kb) *ASGR1* associated with both LDL-cholesterol and each of the outcomes identified [18]. A posterior probability larger than 0.80 provides evidence for colocalization [18]. We set the prior probabilities as recommended, i.e. 1.0e-4 for a variant associated with LDL-cholesterol, 1.0e-4 for a variant associated with the outcome, and 1.0e-5 for a variant associated with both traits [18]. We conducted sensitivity analyses using a prior of 1.0e-6 for a variant associated with both traits, because colocalization results could be sensitive to this choice [25]. We also calculated the posterior probability for a shared variant associated with both traits conditional on the presence of a variant associated with the outcome, as the power to detect colocalization is low when the variants are not strongly associated with the outcome [26].

### Replication

We replicated the findings using other publicly available summary statistics, where possible. We used common (MAF > 1%) variants identified in or near (± 1 Mb) *ASGR1* associated with LDL-cholesterol at genome-wide significance (*p* < 5 × 10^−8^), when rs186021206 or its proxies (*r*^2^ > 0.8) were not available for the outcome GWAS. We expressed genetically mimicked effects of ASGR1 inhibitors in effect sizes of LDL-cholesterol reduction from GLGC (82,587 East Asians) [20] in studies of East Asian ancestry.

### Statistical analysis

We used the *F*-statistic to assess instrument strength for each SNP, approximated by the square of the SNP-exposure association divided by the square of its standard error [27]. An *F*-statistic larger than 10 suggests weak instrument bias is unlikely.

We aligned SNPs on the same allele for exposure and outcome and used proxy SNPs (*r*^2^ > 0.8), where possible, when SNPs were not available in the outcome GWAS. We obtained MR estimates by meta-analysing Wald estimates (genetic association with outcome divided by genetic association with exposure) using inverse variance weighting (IVW) with fixed effects for three SNPs or fewer and random effects for four SNPs or more [28]. We did not give weighted median or MR-Egger estimates for exposures instrumented by correlated SNPs, because their assumptions are unlikely to be satisfied [29].

Differences by sex and between lipid modifiers were assessed using a two-sided *z*-test [30]. We used a statistical significance of 0.05 for all-cause mortality. We used a Benjamini-Hochberg false discovery rate (such that only 5% of significant results are false positive) to correct for multiple comparisons for the 25 *a priori* health outcomes and for the PheWAS rather than the more stringent Bonferroni correction, because the phenotypes investigated are not totally independent [31].

All statistical analyses were conducted using R version 4.2.1 and the packages “TwoSampleMR” for harmonizing data, “MendelianRandomization” for MR analyses, “ieugwasr” for removing correlated SNPs, “coloc” for colocalization analyses, and “metafor” for testing differences by sex and between lipid modifiers. Results were visualized using the packages “ggplot2” and “forestplot”.

## Results

### Genetic mimics of ASGR1 inhibitors and currently used lipid modifiers

We used rs186021206 (MAF 0.46%) to genetically mimic ASGR1 inhibitors. We also used two independent (*r*^2^ < 0.001) common (MAF > 1%) SNPs (rs55714927 and rs150688657) in or near (± 1 Mb) *ASGR1* that are associated with LDL-cholesterol at genome-wide significance (*p* < 5 × 10^−8^) to genetically mimic ASGR1 inhibitors. We used the SNP most strongly associated with LDL-cholesterol (i.e. rs12916 for statins, rs11206510 for PCSK9 inhibitors and rs2073547 (or its proxy rs10260606, *r*^2^ = 0.99) for ezetimibe) in the main analysis and included all relevant SNPs along with their correlations in sensitivity analyses. The F-statistics for the SNPs used to mimic each lipid modifier were all > 10 (Additional file 1: Supplemental Table S4).

### Associations with all-cause mortality

**Fig. 2:**
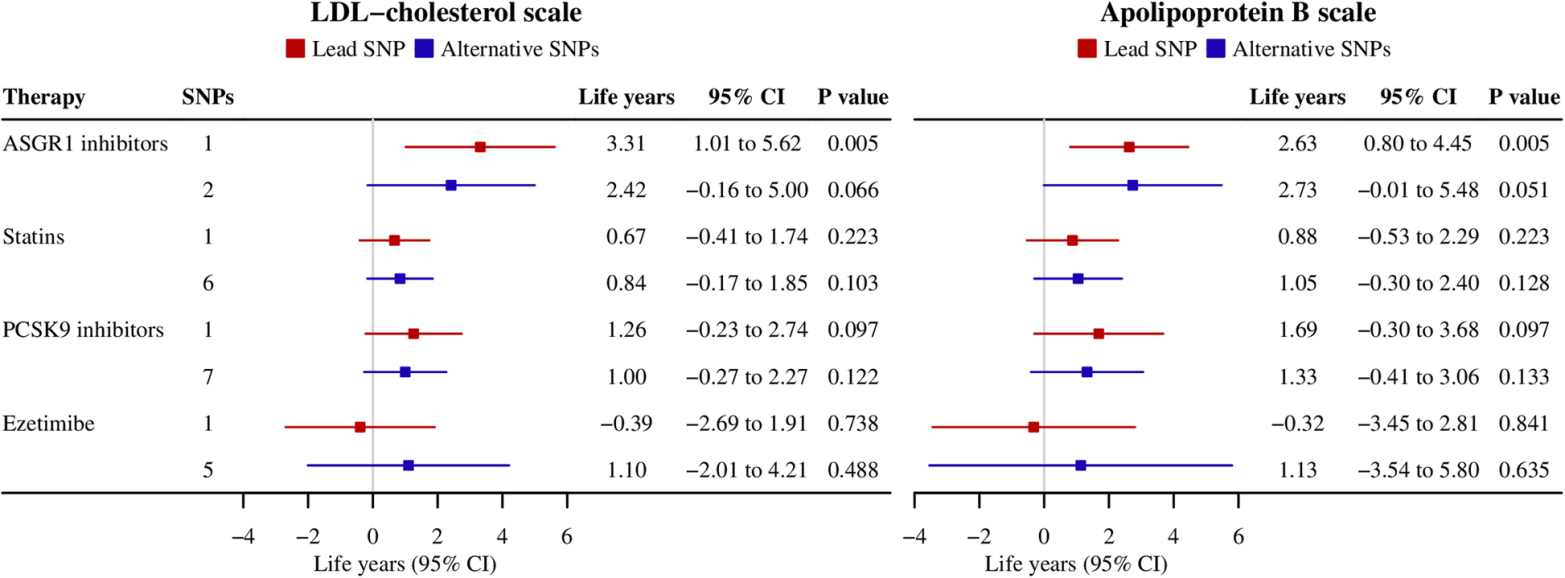
Genetically mimicked effects of ASGR1 inhibitors on lifespan in comparison with currently used lipid modifiers. (a) CI, confidence interval; LDL, low-density lipoprotein. (b) One SNP for ASGR1 inhibitors was rs186021206, and two independent SNPs were rs55714927 and rs150688657; one SNP for statins was rs12916, and six SNPs additionally included rs17238484, rs5909, rs2303152, rs10066707 and rs2006760 along with their correlations; one SNP for PCSK9 inhibitors was rs11206510, and seven SNPs additionally included rs2479409, rs2149041, rs2479394, rs10888897, rs7552841 and rs562556 along with their correlations; one SNP for ezetimibe was rs2073547 (or its proxy rs10260606, *r*^2^ = 0.99), and five SNPs additionally included rs217386, rs7791240, rs10234070 and rs2300414 along with their correlations. (c) Estimates are expressed in life years per standard deviation decrease in LDL-cholesterol or apolipoprotein B

Genetically mimicked ASGR1 inhibitors were associated with longer lifespan (3.31 years per standard deviation reduction in LDL-cholesterol, 95% confidence interval 1.01 to 5.62), whilst the direction for statins and PCSK9 inhibitors was positive but had wide confidence intervals (Fig. 2). Findings were similar in terms of LDL-cholesterol and apoB and using alternative SNPs, i.e. the two independent common SNPs for ASGR1 inhibitors and all relevant SNPs for currently used lipid modifiers along with their correlations (Fig. 2).

### Associations with 25 *a priori* outcomes

**Fig. 3:**
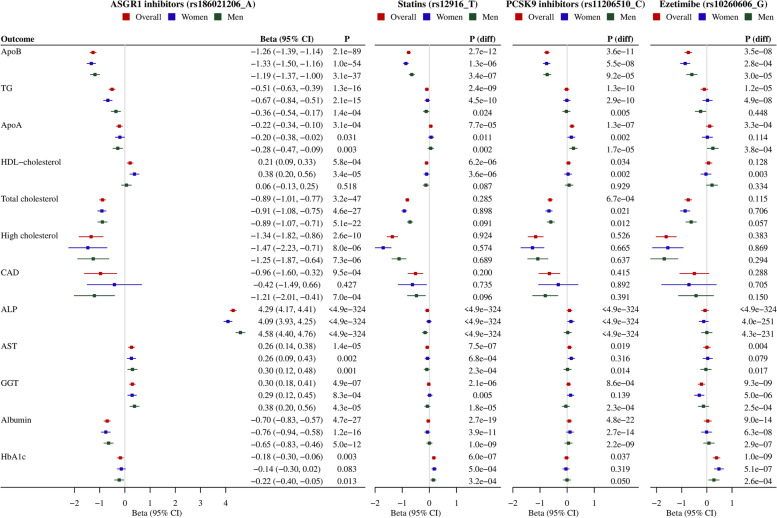
Genetically mimicked effects of ASGR1 inhibitors on the 12 significant outcomes among 25 *a priori* health outcomes in comparison with currently used lipid modifiers. (a) ALP, alkaline phosphatase; ApoA, apolipoprotein A; ApoB, apolipoprotein B; AST, aspartate aminotransferase; CAD, coronary artery disease; GGT, gamma glutamyltransferase; HbA1c, glycated haemoglobin; HDL, high-density lipoprotein; LDL, low-density lipoprotein; TG, triglycerides. (b) P(diff) denotes the *p* value for the comparison of the associations of statins, PCSK9 inhibitors or ezetimibe with those of ASGR1 inhibitors. (c) The G allele of rs10260606 proxied the A allele of rs2073547 (*r*^2^ = 0.99). (d) Estimates are expressed in standard deviation (SD) for continuous outcomes, and in log odds for binary outcomes per SD decrease in LDL-cholesterol

As expected, genetically mimicked ASGR1 inhibitors were associated with lower apoB, TG, apoA, total cholesterol, self-reported high cholesterol risk and CAD risk, and with higher HDL-cholesterol (Fig. 3). These associations were largely similar by sex, despite stronger associations with TG and HDL-cholesterol in women than men (Fig. 3, both *p* values for sex differences 0.01). The associations with apoB and TG were stronger for genetically mimicked ASGR1 inhibitors than currently used lipid modifiers (Fig. 3).

Genetically mimicked ASGR1 inhibitors were associated with higher ALP, AST and GGT, and lower albumin, with a stronger association with ALP in men than women (Fig. 3, *p* value for sex difference < 0.001). These associations were not evident for currently used lipid modifiers (Fig. 3). Genetically mimicked ASGR1 inhibitors had little association with cholelithiasis, adiposity or diabetes (Additional file 1: Supplemental Figure S3), despite an inverse association with HbA1c (Fig. 3). However, genetically mimicked statins and ezetimibe were positively associated with HbA1c (Fig. 3).

### PheWAS

**Fig. 4:**
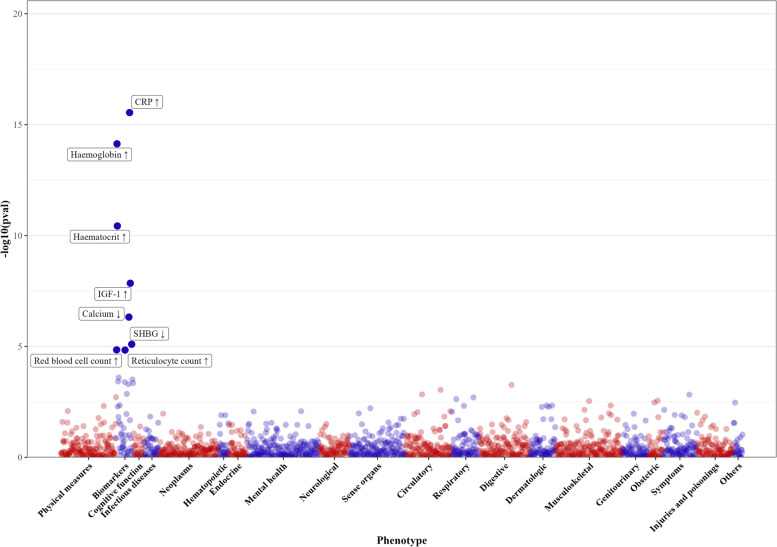
Manhattan plot of genetically mimicked ASGR1 inhibitors (instrumented by the A allele of rs186021206) on 1951 phenotypes in the UK Biobank. (a) CRP, C-reactive protein; IGF-1, insulin-like growth factor 1; SHBG, sex hormone-binding globulin. (b) Each significant phenotype corrected for multiple comparison is highlighted with a label, where ↑ denotes positive association and ↓ denotes negative association

**Fig. 5:**
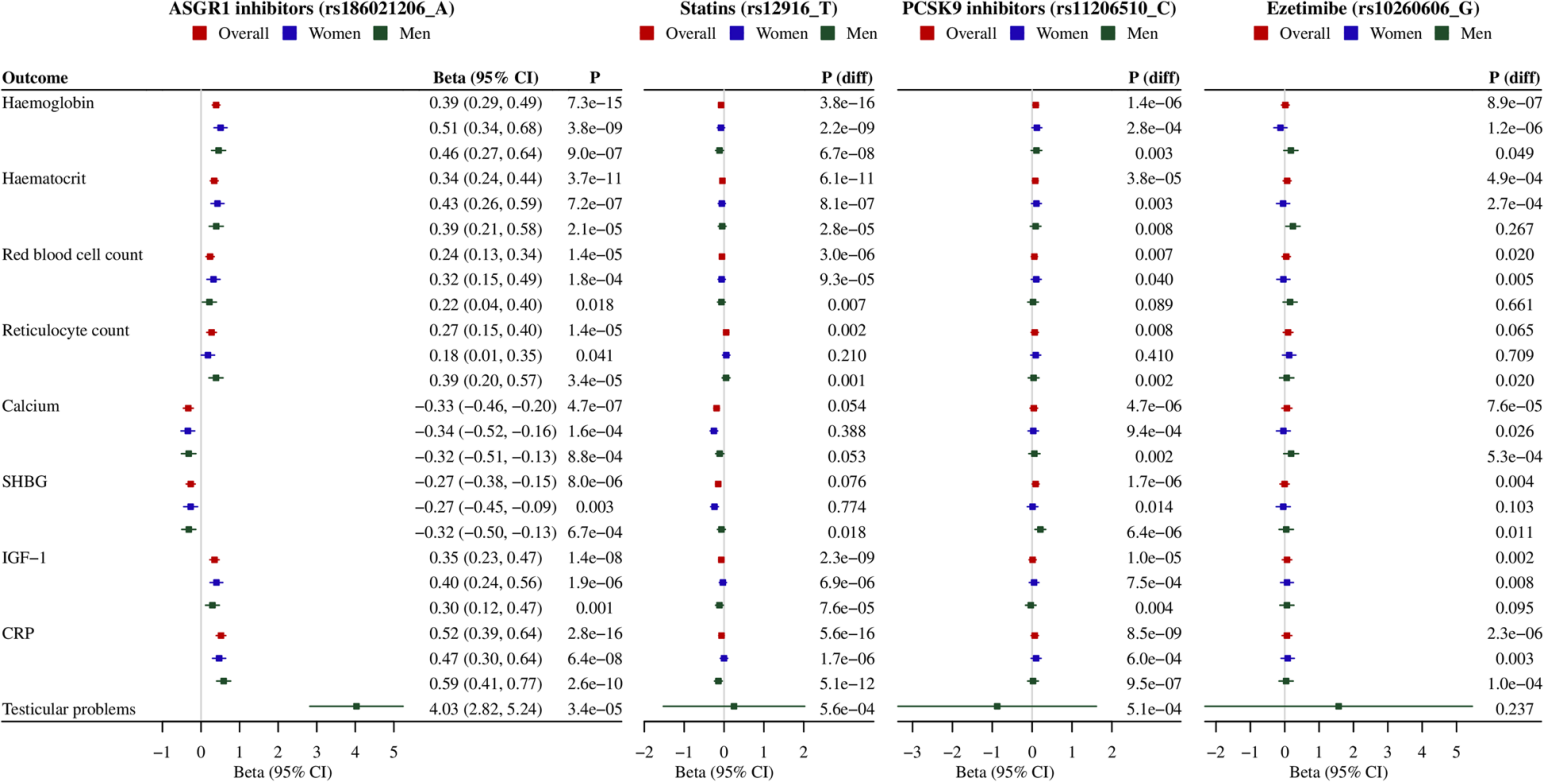
Genetically mimicked effects of ASGR1 inhibitors on health outcomes identified in the phenome-wide association study in comparison with currently used lipid modifiers. (a) CI, confidence interval; CRP, C-reactive protein; IGF-1, insulin-like growth factor 1; SHBG, sex hormone-binding globulin. (b) P(diff) denotes the *p* value for the comparison of the associations of statins, PCSK9 inhibitors or ezetimibe with those of ASGR1 inhibitors. (c) The G allele of rs10260606 proxied the A allele of rs2073547 (*r*^2^ = 0.99). (d) Estimates are expressed in standard deviation (SD) for continuous outcomes, and in log odds for binary outcomes per SD decrease in LDL-cholesterol

After quality control and exclusions, we included 1951 (overall), 1600 (women) and 1523 (men) phenotypes for the PheWAS. A Manhattan plot shows -log10 transformed *p* values for genetically mimicked ASGR1 inhibitors on 1951 phenotypes by category (Fig. 4). After correcting for multiple comparison, genetically mimicked ASGR1 inhibitors were positively associated with erythrocyte traits (haemoglobin concentration, haematocrit percentage, red blood cell count and reticulocyte count), insulin-like growth factor 1 (IGF-1) and C-reactive protein (CRP), and inversely with calcium and sex hormone-binding globulin (SHBG) (Fig. 5). These associations did not differ by sex, and most of them were not evident for currently used lipid modifiers after correction for multiple testing (Fig. 5). However, the inverse associations with calcium and SHBG were also evident for statins specifically in women (Fig. 5, *p* values for sex differences 0.04 and 0.01, respectively). Sex-specific PheWAS did not identify additional phenotypes in women but identified a positive association of genetically mimicked ASGR1 inhibitors with testicular problems in men (Additional file 1: Supplemental Figures S4-5).

### Colocalization analysis

**Fig. 6:**
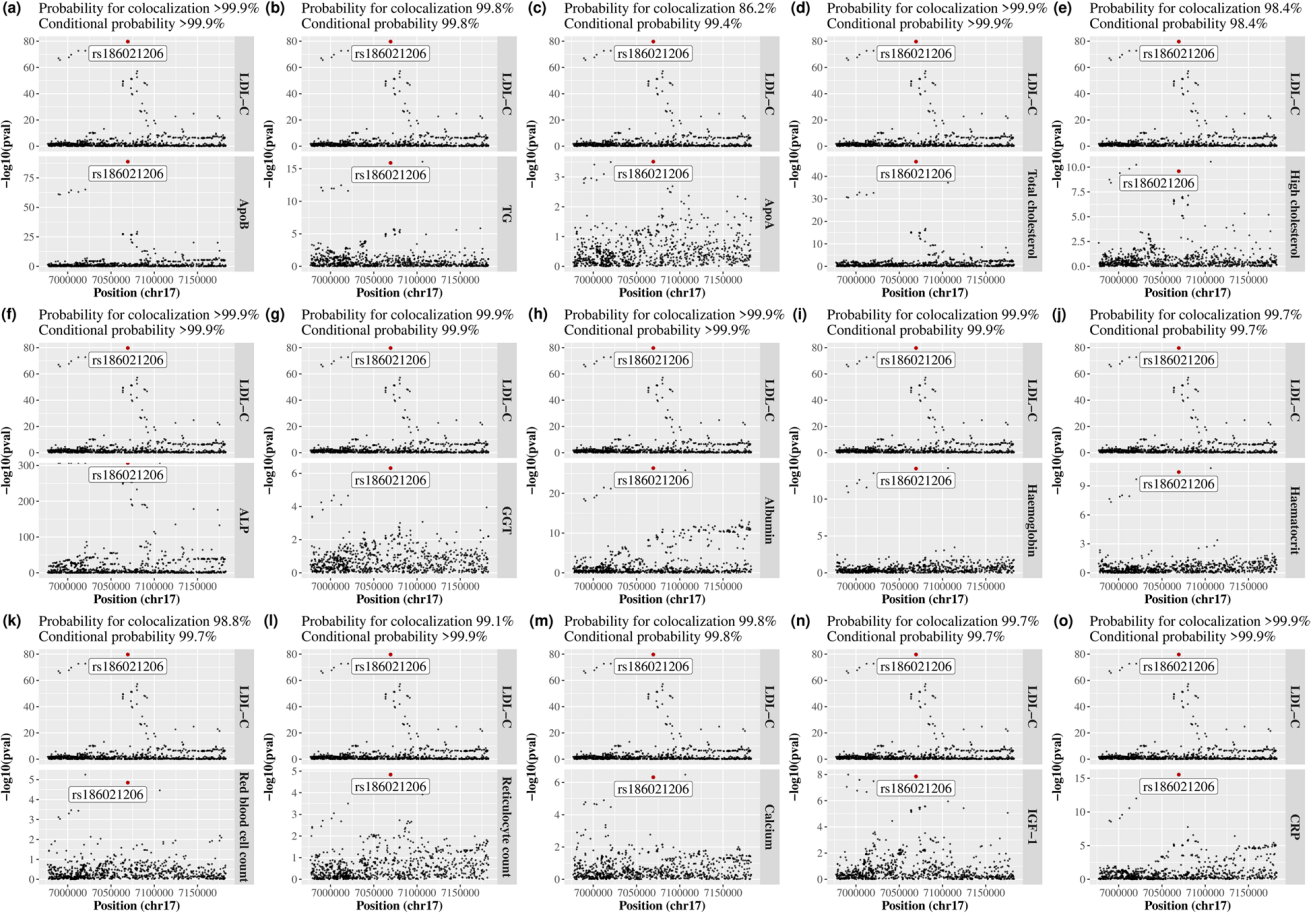
Colocalization analyses for LDL-cholesterol and each significant outcome with probability for colocalization > 0.80 in or near (± 100 kb) the *ASGR1* gene. **a** ALP, alkaline phosphatase; ApoA, apolipoprotein A; ApoB, apolipoprotein B; CRP, C-reactive protein; GGT, gamma glutamyltransferase; IGF-1, insulin-like growth factor 1; LDL, low-density lipoprotein; TG, triglycerides. **b** Prior probabilities were set to 1.0e-4 for a variant associated with LDL-cholesterol, 1.0e-4 for a variant associated with the outcome, and 1.0e-5 for a variant associated with both traits. **c** Probability for colocalization means the posterior probability for a shared variant associated with both traits; conditional probability means the posterior probability for a shared variant associated with both traits conditional on the presence of a variant associated with the outcome. **d** The variant with the largest posterior probability for both traits is highlighted with a label

Colocalization analyses were performed for LDL-cholesterol with each of the significant outcomes. The posterior probabilities for a shared variant with both traits were > 0.80 for most of the significant outcomes (Fig. 6). The posterior probabilities for a shared variant with both traits were < 0.80 for lifespan, HDL-cholesterol, CAD, HbA1c and testicular problems, but were all > 0.80 when conditional on the presence of a variant associated with the outcome (Additional file 1: Supplemental Figure S6). However, the posterior probabilities for a shared variant with both traits were < 0.01, and for two independent variants associated with each trait were > 0.99 for AST and SHBG (Additional file 1: Supplemental Table S5). Colocalization analyses for all significant outcomes consistently identified rs186021206 as the variant with the largest posterior probability for both traits, except the analyses for SHBG which identified rs575551804 (Fig. 6 and Additional file 1: Supplemental Figure S6).

### Replication

For the 21 significant outcomes, most associations for ASGR1 inhibitors were replicated using two independent common *ASGR1* SNPs (rs55714927 and rs150688657), but they had a positive association with SHBG and a possibly inverse association with testicular problems (Additional file 1: Supplemental Table S6). Associations of currently used lipid modifiers with these outcomes were similar when using all relevant SNPs along with their correlations (Additional file 1: Supplemental Table S7).

Associations of genetically mimicked ASGR1 inhibitors with apoB [32], TG [20], apoA1 [32], HDL-cholesterol [20], total cholesterol [20], CAD [33], haemoglobin [34], haematocrit [34], red blood cell count [34], reticulocyte count [35], CRP [36], but not HbA1c [37] were replicated in different GWAS of European ancestry, and associations with liver function (ALP, AST, GGT and albumin) [38] and calcium [38] were replicated in GWAS of East Asians (Additional file 1: Supplemental Table S8).

## Discussion

Consistent with previous studies [2, 3], we found genetically mimicked ASGR1 inhibitors associated with lower apoB, TG, total cholesterol and CAD risk. Our study adds by providing novel genetic evidence suggesting ASGR1 inhibitors reduce all-cause mortality, identifying non-lipid effects of ASGR1 inhibitors on liver function, erythrocyte traits, calcium, IGF-1 and CRP and confirming our findings using colocalization and replication.

Genetically mimicked ASGR1 inhibitors were positively associated with lifespan, possibly outperforming currently used lipid modifiers (Fig. 2). Correspondingly, a previous genetic analysis showed the del12 mutation in *ASGR1* has a greater magnitude of effect on CAD risk than other variants lowering non-HDL-cholesterol [2]. These differences may be related to ASGR1 inhibitors reducing apoB and TG more than currently used lipid modifiers. Alternatively, other mechanisms may play a role, for example, adverse effects on weight gain and type 2 diabetes risk could detract from beneficial effects of statins on lifespan [9].

When examining associations of genetically mimicked ASGR1 inhibitors with the 25 *a priori* outcomes, we found an association with higher ALP, consistent with previous MR studies [2, 3]. ALP is a glycoprotein known to bind ASGPR, and thus inhibiting ASGR1 decreases the clearance of ALP from the circulation [39]. Previous studies suggested ASGR1 deficiency is associated with higher ALT, AST and GGT in pigs [6], but a loss-of-function *ASGR1* variant has little association with AST, ALT and bilirubin in humans although a mild increase in GGT and decrease in albumin cannot be excluded [2]. Using a large sample to increase statistical power, we showed genetically mimicked ASGR1 inhibitors were associated with higher GGT and lower albumin, which was supported by colocalization. These findings suggest a potentially adverse effect of ASGR1 inhibitors on liver function. However, effects of liver function on CAD seem limited [40–42], although higher GGT and albumin may increase CAD risk [41, 42]. Mild-to-moderate elevations in aminotransferase are common in statin users, but statin-induced liver injury is rare even for those with elevated baseline liver enzymes [43, 44]. We did not find an association of genetically mimicked ASGR1 inhibitors with cholelithiasis, in contrast to a previous hypothesis that inhibiting ASGR1 upregulates ABCG5/8 and subsequently promotes cholelithiasis [4, 7].

In the PheWAS, we found genetically mimicked ASGR1 inhibitors were positively associated with erythrocyte traits, IGF-1 and CRP, but inversely with calcium. Previous MR studies suggest that higher reticulocyte count and possibly haemoglobin, haematocrit and red blood cell count increase CAD risk [35, 42, 45]; higher IGF-1 increases the risk of CAD and some cancers [46–49]; and CRP has a neutral role in CAD, cancer and lifespan [50–52], whilst lower calcium decreases CAD risk and increases lifespan [53, 54]. An inverse association of genetically mimicked statins with calcium has also been reported [13]. Given the strong associations of genetically mimicked ASGR1 inhibitors with lower CAD risk and longer lifespan, these non-lipid effects would appear to be mainly of etiological interest.

Non-lipid effects of ASGR1 inhibitors generally differed from those of currently used lipid modifiers. Notably, genetic mimics of ASGR1 inhibitors were not associated with the higher BMI or type 2 diabetes risk seen for statins [9], possibly because of different mechanisms. Statins inhibit cholesterol synthesis via 3-hydroxy-3-methylglutaryl–coenzyme A reductase (HMGCR), PCSK9 inhibitors increase LDL-receptors, and ezetimibe decreases cholesterol absorption [55]. ASGR1 inhibitors decrease cholesterol synthesis by downregulating HMGCR and increase cholesterol clearance by upregulating LDL-receptors [6, 56]. However, ASGR1 inhibitors also reduces lipogenesis by activating adenosine monophosphate (AMP)-activated protein kinase (AMPK) and thereby inhibiting sterol regulatory element-binding protein 1 (SREBP1) [4]. AMPK plays an essential role in cellular energy homeostasis [57], which may offset any detrimental effects of inhibiting HMGCR on BMI and type 2 diabetes [9]. AMPK is also involved in the regulation of erythrocyte survival [58], which might explain the effects of ASGR1 inhibitors on erythrocyte traits. ASGR1 inhibitors promote cholesterol excretion by upregulating liver X receptor α [4], which may cause hepatic steatosis and elevate liver enzymes [59]. It is also possible that endoplasmic reticulum stress-induced hepatocyte apoptosis drives the potentially adverse effect of ASGR1 inhibitors on liver function [6].

Colocalization analysis identified rs186021206 as the SNP with the largest posterior probability for both LDL-cholesterol and CAD, which substantiates its use as a genetic mimic of ASGR1 inhibitors. Colocalization generally substantiated the findings, although the posterior probabilities were < 0.80 for lifespan, HDL-cholesterol and CAD, probably due to insufficient power given the conditional posterior probabilities for colocalization were all > 0.80 [26]. However, the posterior probabilities for two independent variants associated with each trait were > 0.99 for AST and SHBG, suggesting the associations of genetically mimicked ASGR1 inhibitors with AST and SHBG could be confounded by linkage disequilibrium [18].

This is the first study comprehensively investigating genetically mimicked effects of ASGR1 inhibitors in comparison with currently used lipid modifiers on lifespan and a range of potentially relevant health outcomes substantiated by an agnostic search for novel effects and colocalization. Nevertheless, this study has several limitations. First, MR should fulfil the instrumental variable assumptions of relevance, independence and exclusion restriction, that is genetic instruments should be strongly related to the exposure, share no common cause with the outcome and be independent of the outcome given the exposure [8]. To satisfy the relevance assumption, we checked the *F*-statistics for all the SNPs were > 10, suggesting weak instrument bias was unlikely. We used well-established, functionally relevant SNPs to mimic each lipid modifier to reduce the possibility of pleiotropic effects on the outcomes through pathways unrelated to the drug targets [60]. We expressed the effects of each lipid modifier in effect sizes of LDL-cholesterol reduction. This presentation does not imply that any consequences of lipid modifiers work through LDL-cholesterol but provides an interpretable means of quantifying the MR estimates for comparability. The small number of independent genetic mimics for each lipid modifier considered precluded the use of pleiotropy robust MR methods and limited the power to identify potential effects of ASGR1 inhibitors. We used colocalization to assess the validity of the genetic mimic and any associations found for ASGR1 inhibitors. However, we cannot exclude the possibility that some effects of ASGR1 inhibitors have been missed. Second, the *ASGR1*, *HMGCR*, *PCSK9* and *NPC1L1* variants may affect the prescription of lipid modifiers and thus mitigate their genetic effects on lifespan. However, such complementary mechanisms would not explain the positive associations of genetically mimicked ASGR1 inhibitors with lifespan. Third, PheWAS is comprehensive but agnostic. Nevertheless, it provides insights about unknown effects of ASGR1 inhibitors, which has implications for drug development including identifying potential side-effects and elucidating mechanisms. Replication using other large GWAS excluding UK Biobank participants would be worthwhile, when available. Fourth, genetic associations for binary phenotypes were obtained using linear regression in the UK Biobank (http://www.nealelab.is/uk-biobank/), which can inflate false positives when the case number is small and the SNP MAF is rare. However, results were validated using two common *ASGR1* variants. Fifth, MR could be open to selection bias, particularly from recruiting survivors [61]. However, the UK Biobank participants were relatively young likely obviating selective survival to recruitment on genetic endowment for CAD. We used parental attained age as a measure of all-cause mortality, which reduces selection bias from only recruiting survivors. Sixth, associations in people of European ancestry may not apply to other populations. However, causal effects should act consistently across settings unless the mediating mechanisms differ [62], for example, genetically mimicked effects of ASGR1 inhibitors on liver function were replicated in East Asians. Finally, MR assesses the lifelong effects of inhibiting ASGR1 which may not directly reflect quantitative effects of ASGR1 inhibitors in the short term. Further investigation is needed to confirm these findings in clinical practice.

## Conclusions

Our MR study provides genetic evidence that ASGR1 inhibitors may reduce all-cause mortality, comparing favourably with currently used lipid modifiers. Beyond lipid-lowering, genetically mimicked ASGR1 inhibitors increased liver enzymes, erythrocyte traits, IGF-1 and CRP, but decreased albumin and calcium. These insights highlight ASGR1 as a promising therapeutic target for reducing CAD morbidity and mortality and prioritize further investigation of non-lipid pathways underlying the health effects of ASGR1 inhibitors.

## Supplementary Information

**Additional file 1: Fig. S1.** Flowchart of the study design. **Fig. S2.** Flowchart of phenotype selection for the phenome-wide association study using the UK Biobank summary statistics provided by Neale lab. **Fig. S3.** Genetically mimicked effects of ASGR1 inhibitors on the 13 non-significant outcomes among 25 *a priori* health outcomes in comparison with currently used lipid modifiers. **Fig. S4.** Manhattan plot of genetically mimicked ASGR1 inhibitors on 1600 phenotypes for women in the UK Biobank. **Fig. S5.** Manhattan plot of genetically mimicked ASGR1 inhibitors on 1523 phenotypes for men in the UK Biobank. **Fig. S6.** Colocalization analyses for LDL-cholesterol and each significant outcome with probability for colocalization <0.80 in or near the *ASGR1* gene. **Tab. S1.** Correlation matrix of genetic mimics of statins. **Tab. S2.** Correlation matrix of genetic mimics of PCSK9 inhibitors. **Tab. S3.** Correlation matrix of genetic mimics of ezetimibe. **Tab. S4.** SNP-specific estimates for genetically mimicked ASGR1 inhibitors, statins, PCSK9 inhibitors and ezetimibe on LDL-cholesterol from GLGC excluding UK Biobank participants. **Tab. S5.** Colocalization estimates for each posterior probability using prior probabilities 1.0E-4 for a variant associated with LDL-cholesterol, 1.0E-4 for a variant associated with the outcome, and different values for a variant associated with both traits in or near the *ASGR1* gene. **Tab. S6.** Mendelian randomization inverse variance weighted estimates for genetically mimicked ASGR1 inhibitors on significant outcomes. **Tab. S7.** Mendelian randomization inverse variance weighted estimates for genetically mimicked statins, PCSK9 inhibitors and ezetimibe on significant outcomes identified for ASGR1 inhibitors. **Tab. S8.** Mendelian randomization inverse variance weighted estimates for genetically mimicked ASGR1 inhibitors on significant outcomes in replication studies. **Supplemental Note.** R code for Mendelian randomization analysis, phenome-wide association analysis and colocalization analysis.

**Additional file 2: Tab. S9.** Associations of genetically mimicked ASGR1 inhibitors with 1951 phenotypes for both sexes in the UK Biobank. **Tab. S10.** Associations of genetically mimicked ASGR1 inhibitors with 1600 phenotypes for women in the UK Biobank. **Tab. S11.** Associations of genetically mimicked ASGR1 inhibitors with 1523 phenotypes for men in the UK Biobank.

## Data Availability

Summary-level data analysed during the current study are available from the website http://www.nealelab.is/uk-biobank/ for UK Biobank (Neale lab), https://pheweb.jp/downloads for Biobank Japan, http://csg.sph.umich.edu/willer/public/glgc-lipids2021/ for GLGC, http://www.cardiogramplusc4d.org/data-downloads/ for CARDIoGRAMplusC4D, https://magicinvestigators.org/downloads/ for Meta-Analyses of Glucose and Insulin-related traits Consortium (MAGIC) and https://www.ebi.ac.uk/gwas/ for other GWAS. R code for data analysis is shown in Additional file 1: Supplemental Note. Results for the PheWAS of genetically mimicked ASGR1 inhibitors on 1951 (overall), 1600 (women) and 1523 (men) phenotypes in the UK Biobank are shown in Additional file 2: Supplemental Tables S9-11.

## Abbreviations

ABCG5/8: Adenosine triphosphate-binding cassette transporters G5/8
ALP: Alkaline phosphatase
ALT: Alanine transaminase
AMPK: Adenosine monophosphate (AMP)-activated protein kinase
ApoA: Apolipoprotein A
ApoB: Apolipoprotein B
ASGR1: Asialoglycoprotein receptor 1
AST: Aspartate aminotransferase
BMI: Body mass index
CAD: Coronary artery disease
CRP: C-reactive protein
CVD: Cardiovascular disease
GGT: Gamma glutamyltransferase
GLGC: Global Lipids Genetics Consortium
GWAS: Genome-wide association study
HbA1c: Glycated haemoglobin
HDL: High-density lipoprotein
HMGCR: 3-Hydroxy-3-methylglutaryl–coenzyme A reductase
ICD: International Classification of Disease
IGF-1: Insulin-like growth factor 1
IVW: Inverse variance weighting
LDL: Low-density lipoprotein
Lp(a): Lipoprotein(a)
MAF: Minor allele frequency
MAGIC: Meta-Analyses of Glucose and Insulin-related traits Consortium
MR: Mendelian randomization
PCSK9: Proprotein convertase subtilisin/kexin type 9
PheWAS: Phenome-wide association study
SHBG: Sex hormone-binding globulin
SREBP1: Sterol regulatory element-binding protein 1
TG: Triglycerides
