# Supplementary material for: Genetically mimicked effects of ASGR1 inhibitors on all-cause mortality and health outcomes: a drug-target Mendelian randomization study and a phenome-wide association study
Source: BMC Med. 2023 Jul 3;21:235. doi: 10.1186/s12916-023-02903-w (PMC10318778; doi:10.1186/s12916-023-02903-w)
Supplement: Supplementary file 1 — Additional file 1: Fig. S1. Flowchart of the study design. Fig. S2. Flowchart of phenotype selection for the phenome-wide association study using the UK Biobank summary statistics provided by Neale lab. Fig. S3. Genetically mimicked effects of ASGR1 inhibitors on the 13 non-significant outcomes among 25 a priori health outcomes in comparison with currently used lipid modifiers. Fig. S4. Manhattan plot of genetically mimicked ASGR1 inhibitors on 1600 phenotypes for women in the UK Biobank. Fig. S5. Manhattan plot of genetically mimicked ASGR1 inhibitors on 1523 phenotypes for men in the UK Biobank. Fig. S6. Colocalization analyses for LDL-cholesterol and each significant outcome with probability for colocalization <0.80 in or near the ASGR1 gene. Tab. S1. Correlation matrix of genetic mimics of statins. Tab. S2. Correlation matrix of genetic mimics of PCSK9 inhibitors. Tab. S3. Correlation matrix of genetic mimics of ezetimibe. Tab. S4. SNP-specific estimates for genetically mimicked ASGR1 inhibitors, statins, PCSK9 inhibitors and ezetimibe on LDL-cholesterol from GLGC excluding UK Biobank participants. Tab. S5. Colocalization estimates for each posterior probability using prior probabilities 1.0E-4 for a variant associated with LDL-cholesterol, 1.0E-4 for a variant associated with the outcome, and different values for a variant associated with both traits in or near the ASGR1 gene. Tab. S6. Mendelian randomization inverse variance weighted estimates for genetically mimicked ASGR1 inhibitors on significant outcomes. Tab. S7. Mendelian randomization inverse variance weighted estimates for genetically mimicked statins, PCSK9 inhibitors and ezetimibe on significant outcomes identified for ASGR1 inhibitors. Tab. S8. Mendelian randomization inverse variance weighted estimates for genetically mimicked ASGR1 inhibitors on significant outcomes in replication studies. Supplemental Note. R code for Mendelian randomization analysis, phenome-wide association analysis and colocal [file 12916_2023_2903_MOESM1_ESM.pdf]

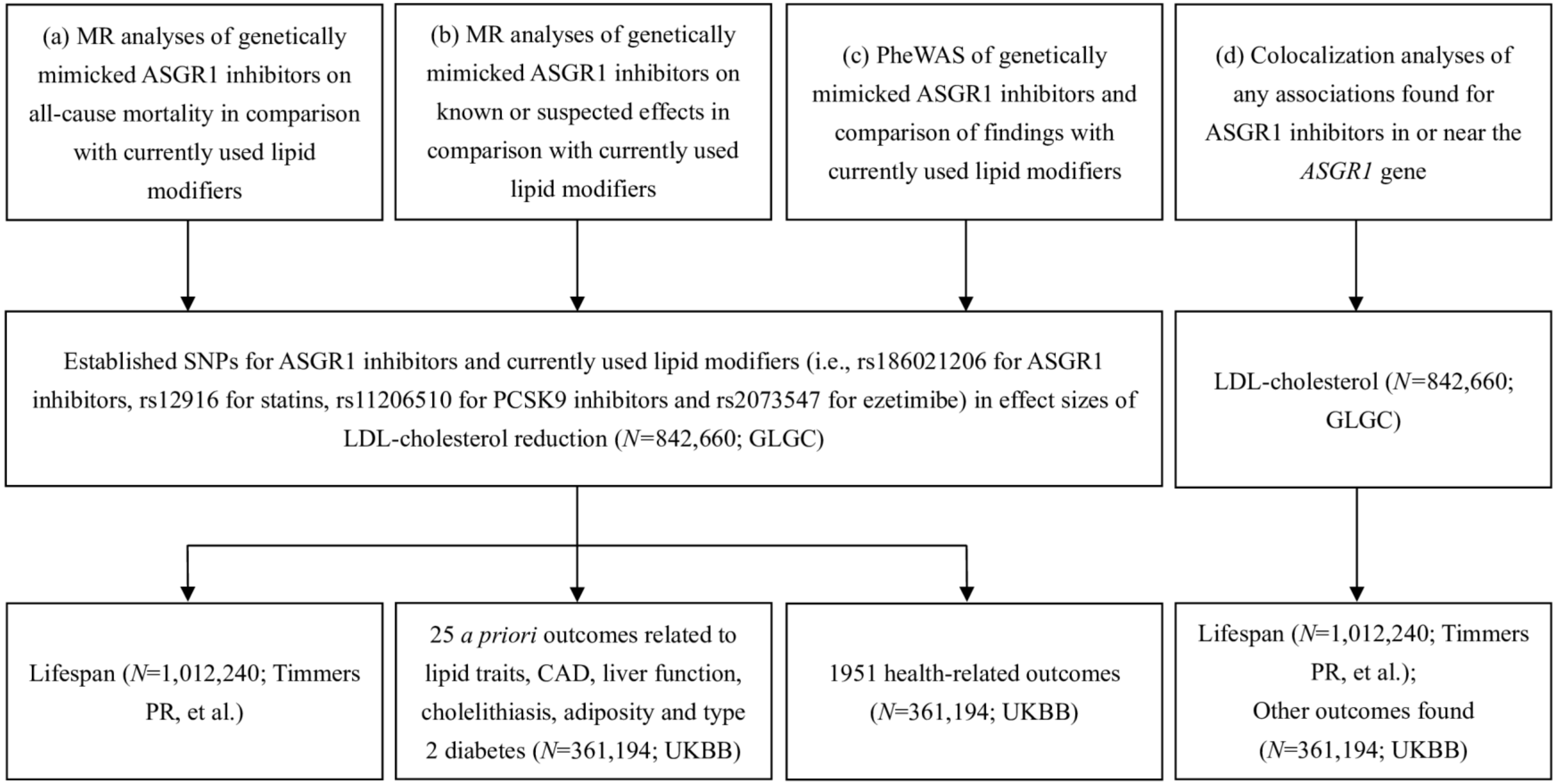

Supplemental Figure S1. Flowchart of the study design.

a. CAD, coronary artery disease; GLGC, Global Lipids Genetics Consortium; MR, Mendelian randomization; PheWAS, phenome-wide association study; UKBB, UK Biobank.

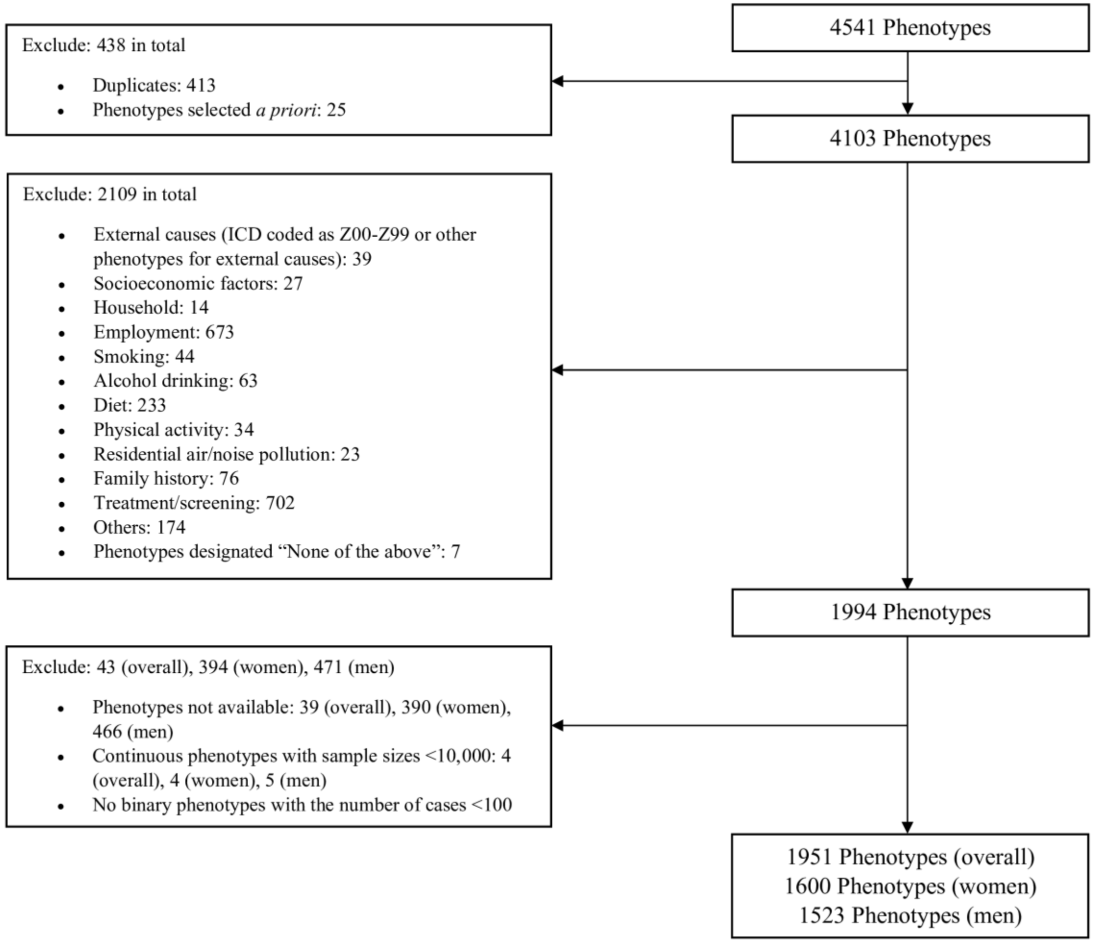

Others: Age, sex, reception, hospital administration, questionnaire/test completion, reaction time, skin/hair colour, congenital malformation, early life factors, sexual orientation, social activities, sun exposure, addiction, phone use, glasses wearing, heating, transportation, age at disease onset or diagnosis, age at death and underlying cause of death.

Supplemental Figure S2. Flowchart of phenotype selection for the phenome-wide association study using the UK Biobank summary statistics provided by Neale lab.

a. ICD, International Classification of Disease.

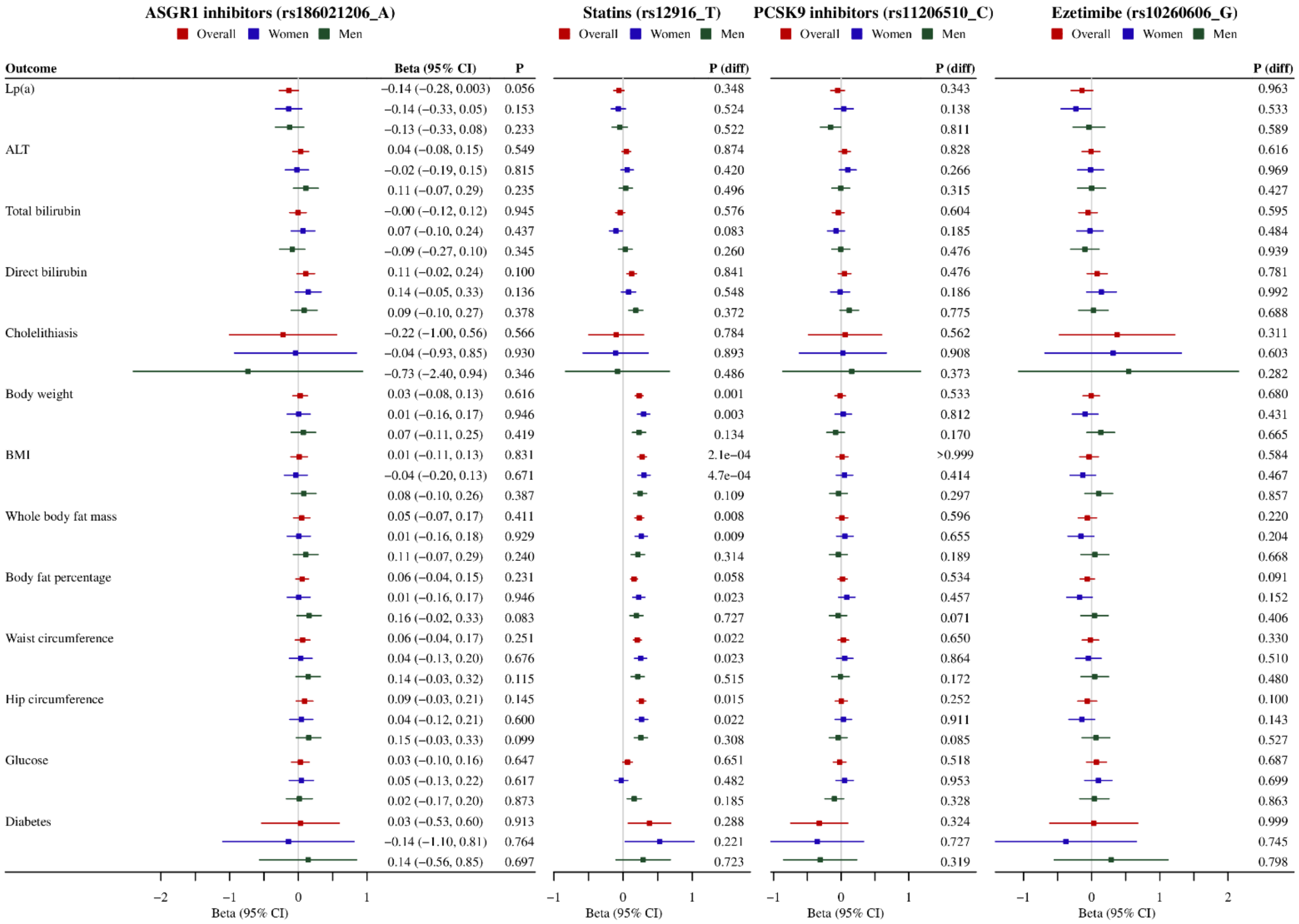

Supplemental Figure S3. Genetically mimicked effects of ASGR1 inhibitors on the 13 non-significant outcomes among 25 *a priori* health outcomes in comparison with currently used lipid modifiers.

a. ALT, alanine transaminase; BMI, body mass index; CI, confidence interval; Lp(a), lipoprotein(a).

b. P(diff) denotes the *p* value for the comparison of the associations of statins, PCSK9 inhibitors or ezetimibe with those of ASGR1 inhibitors.

c. The G allele of rs10260606 proxied the A allele of rs2073547 (r^2=0.99).

d. Estimates are expressed in standard deviation (SD) for continuous outcomes, and in log odds for binary outcomes per SD decrease in LDL-cholesterol.

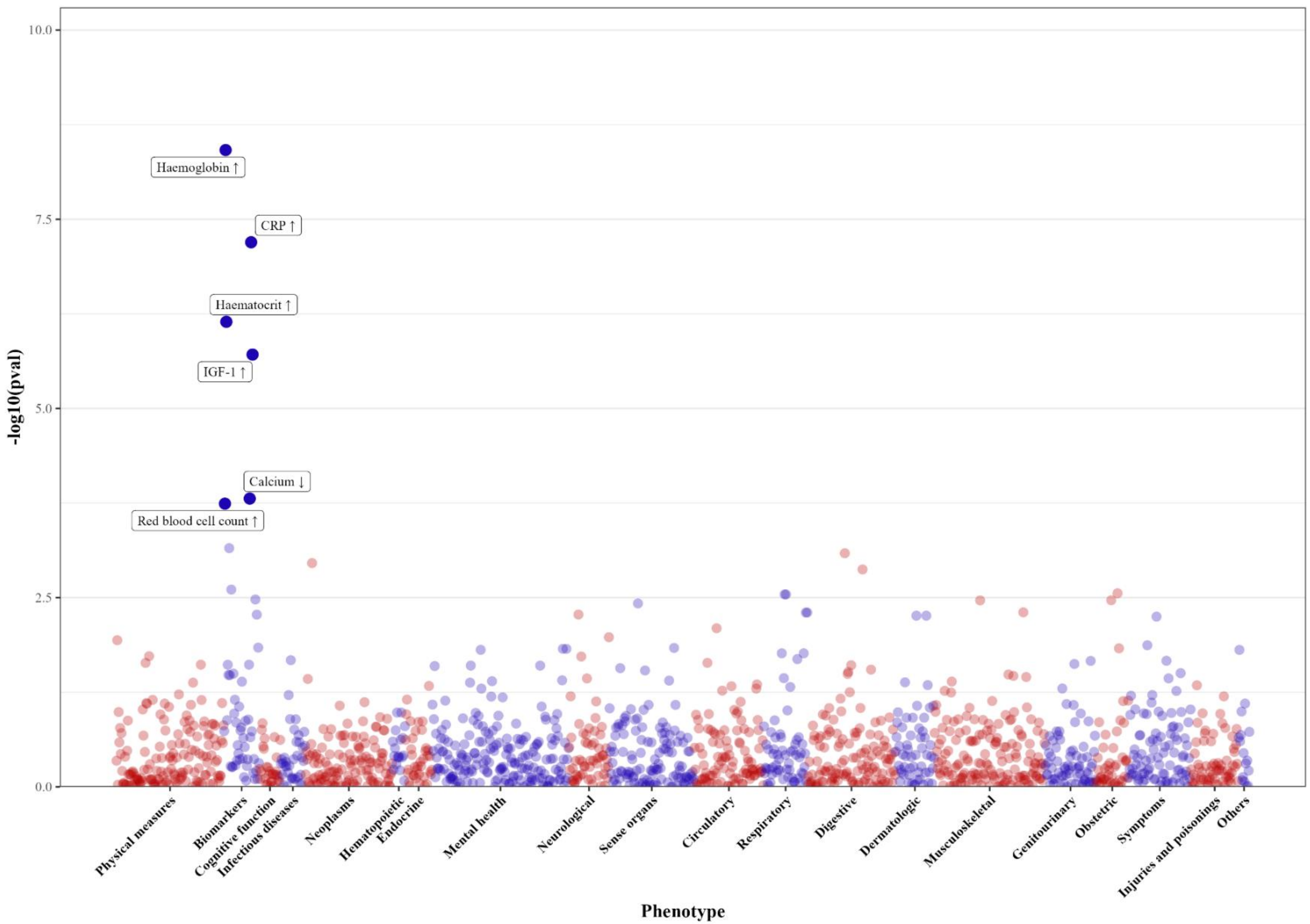

Supplemental Figure S4. Manhattan plot of genetically mimicked ASGR1 inhibitors (instrumented by the A allele of rs186021206) on 1600 phenotypes for women in the UK Biobank.

a. CRP, C-reactive protein; IGF-1, insulin-like growth factor 1.

b. Each significant phenotype corrected for multiple comparison is highlighted with a label, where ↑ denotes positive association and ↓ denotes negative association.

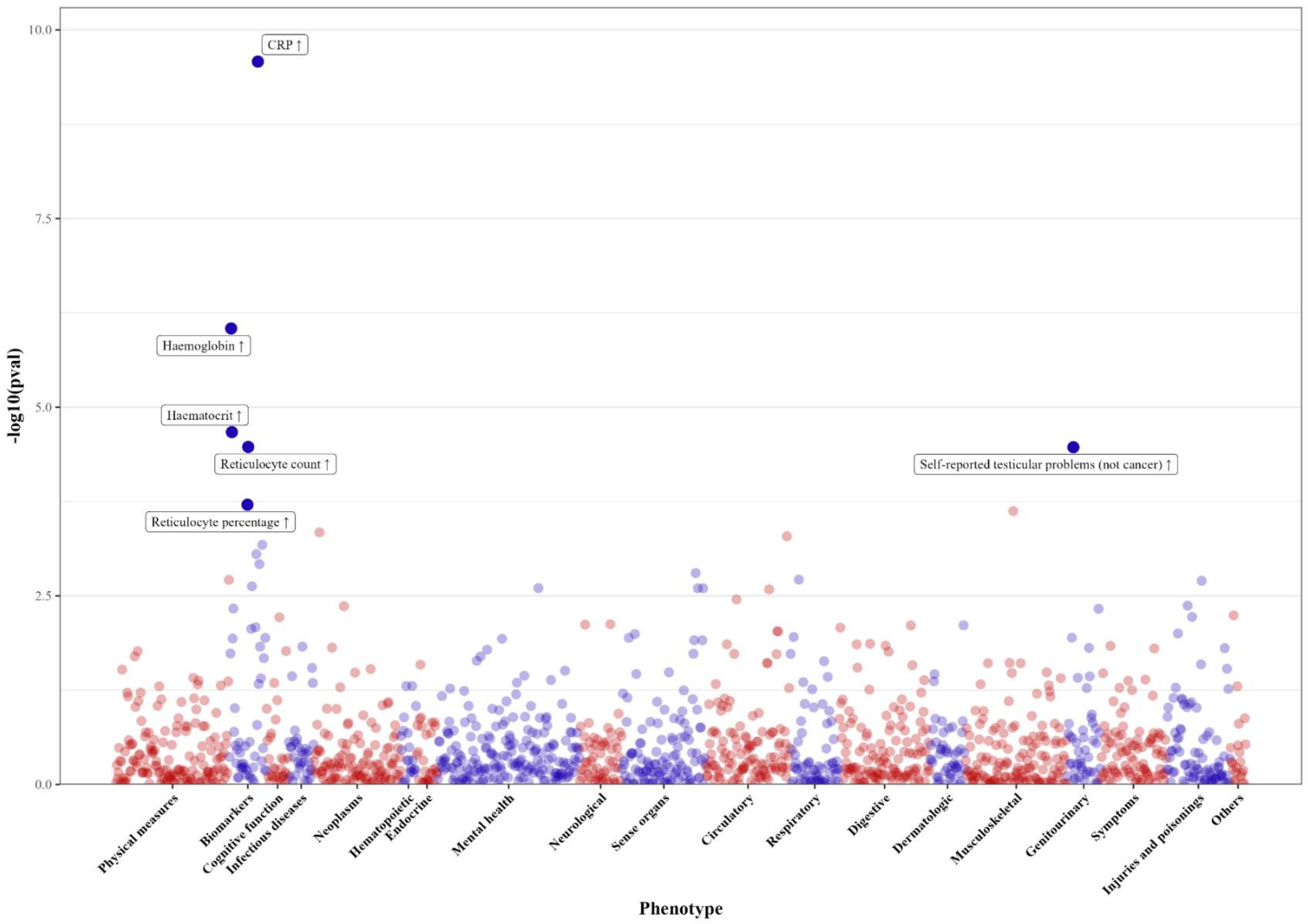

Supplemental Figure S5. Manhattan plot of genetically mimicked ASGR1 inhibitors (instrumented by the A allele of rs186021206) on 1523 phenotypes for men in the UK Biobank.

a. CRP, C-reactive protein.

b. Each significant phenotype corrected for multiple comparison is highlighted with a label, where ↑ denotes positive association and ↓ denotes negative association.

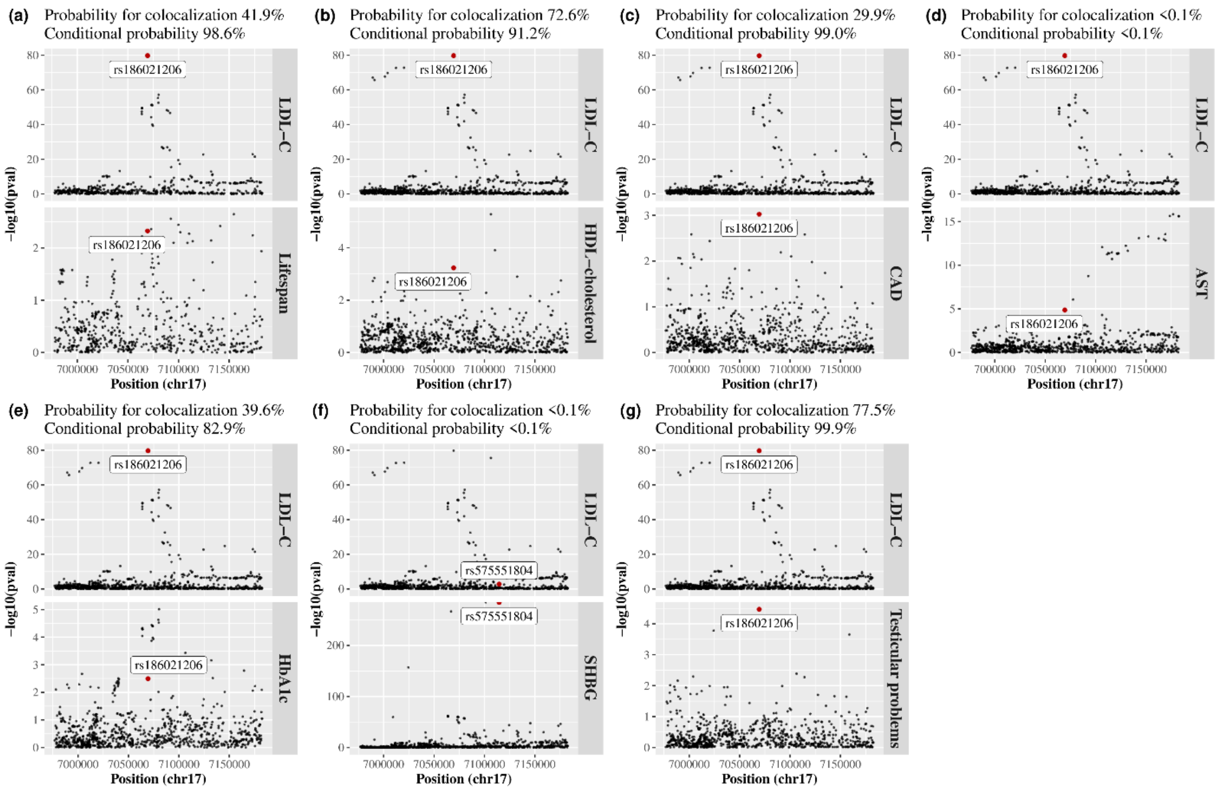

Supplemental Figure S6. Colocalization analyses for LDL-cholesterol and each significant outcome with probability for colocalization <0.80 in or near (+-100kb) the *ASGR1* gene.

a. AST, aspartate aminotransferase; CAD, coronary artery disease; HbA1c, glycated haemoglobin; HDL, high-density lipoprotein; LDL, low-density lipoprotein; SHBG, sex hormone-binding globulin.

b. Prior probabilities were set to 1.0e-4 for a variant associated with LDL-cholesterol, 1.0e-4 for a variant associated with the outcome, and 1.0e-5 for a variant associated with both traits.

c. Probability for colocalization means the posterior probability for a shared variant associated with both traits; conditional probability means the posterior probability for a shared variant associated with both traits conditional on the presence of a variant associated with the outcome.

d. The variant with the largest posterior probability for both traits is highlighted with a label.

Supplemental Table S1: Correlation matrix of genetic mimics of statins.

Correlation coefficient	rs12916_C_T	rs17238484_T_G	rs5909_A_G	rs2303152_A_G	rs10066707_A_G	rs2006760_G_C
rs12916_C_T	1.00	0.67	0.36	0.44	0.50	0.40
rs17238484_T_G	0.67	1.00	-0.23	0.64	0.54	0.24
rs5909_A_G	0.36	-0.23	1.00	-0.13	0.21	0.45
rs2303152_A_G	0.44	0.64	-0.13	1.00	0.28	0.52
rs10066707_A_G	0.50	0.54	0.21	0.28	1.00	0.48
rs2006760_G_C	0.40	0.24	0.45	0.52	0.48	1.00

Supplemental Table S2: Correlation matrix of genetic mimics of PCSK9 inhibitors.

Correlation coefficient	rs11206510_C_T	rs2479409_G_A	rs2149041_G_C	rs2479394_G_A	rs10888897_T_C	rs7552841_T_C	rs562556_G_A
rs11206510_C_T	1.00	-0.20	-0.16	-0.30	0.24	0.12	0.15
rs2479409_G_A	-0.20	1.00	0.61	0.20	-0.34	-0.03	-0.19
rs2149041_G_C	-0.16	0.61	1.00	0.25	-0.28	-0.07	-0.18
rs2479394_G_A	-0.30	0.20	0.25	1.00	-0.08	-0.13	-0.02
rs10888897_T_C	0.24	-0.34	-0.28	-0.08	1.00	0.08	0.33
rs7552841_T_C	0.12	-0.03	-0.07	-0.13	0.08	1.00	-0.23
rs562556_G_A	0.15	-0.19	-0.18	-0.02	0.33	-0.23	1.00

Supplemental Table S3: Correlation matrix of genetic mimics of ezetimibe.

Correlation coefficient	rs217386_A_G	rs2073547_G_A	rs7791240_C_T	rs10234070_T_C	rs2300414_A_G
rs217386_A_G	1.00	-0.38	-0.25	-0.20	-0.22
rs2073547_G_A	-0.38	1.00	0.54	0.46	0.30
rs7791240_C_T	-0.25	0.54	1.00	0.15	0.60
rs10234070_T_C	-0.20	0.46	0.15	1.00	0.15
rs2300414_A_G	-0.22	0.30	0.60	0.15	1.00

Supplemental Table S4. SNP-specific estimates for genetically mimicked ASGR1 inhibitors, statins, PCSK9 inhibitors and ezetimibe on LDL-cholesterol (standard deviation) from GLGC excluding UK Biobank participants.

Therapy	SNP	Effect allele	Other allele	Effect allele frequency	Beta	SE	*P* value	F-statistic
ASGR1 inhibitors	rs186021206	A	G	0.005	-0.2552	0.0134	6.3E-63	360.7
ASGR1 inhibitors	rs55714927	T	C	0.187	-0.0352	0.0022	2.1E-45	257.3
ASGR1 inhibitors	rs150688657	A	G	0.103	0.0212	0.0027	9.9E-12	59.6
Statins	rs12916	C	T	0.403	0.0716	0.0017	1.94e-314	1849.1
Statins	rs10066707	A	G	0.374	0.0507	0.0018	3.4E-143	835.3
Statins	rs17238484	T	G	0.226	0.0606	0.0020	8.0E-164	957.4
Statins	rs2006760	G	C	0.203	0.0484	0.0021	4.3E-90	521.2
Statins	rs2303152	A	G	0.096	0.0381	0.0028	4.4E-34	190.6
Statins	rs5909	A	G	0.099	0.0553	0.0027	4.7E-72	414.7
PCSK9 inhibitors	rs11206510	C	T	0.179	-0.0658	0.0021	3.7E-160	935.7
PCSK9 inhibitors	rs10888897	T	C	0.374	-0.0393	0.0018	8.6E-87	501.7
PCSK9 inhibitors	rs2149041	G	C	0.178	0.0496	0.0021	4.6E-93	538.8
PCSK9 inhibitors	rs2479394	G	A	0.282	0.0288	0.0018	4.0E-45	255.7
PCSK9 inhibitors	rs2479409	G	A	0.338	0.0475	0.0018	1.3E-124	725.4
PCSK9 inhibitors	rs562556	G	A	0.173	-0.0447	0.0022	1.8E-74	428.9
PCSK9 inhibitors	rs7552841	T	C	0.364	0.0345	0.0020	2.8E-53	303.8
Ezetimibe	rs2073547	G	A	0.202	0.0427	0.0021	3.9E-71	409.2
Ezetimibe	rs10234070	T	C	0.122	0.0215	0.0026	1.1E-13	71.0
Ezetimibe	rs217386	A	G	0.419	-0.0307	0.0017	1.5E-57	328.9
Ezetimibe	rs2300414	A	G	0.077	0.0248	0.0031	1.6E-12	64.2
Ezetimibe	rs7791240	C	T	0.098	0.0334	0.0028	3.7E-26	144.0

a. GLGC, Global Lipids Genetics Consortium; LDL, low-density lipoprotein.

Supplemental Table S5. Colocalization estimates for each posterior probability using prior probabilities 1.0E-4 for a variant associated with LDL-cholesterol, 1.0E-4 for a variant associated with the outcome, and different values for a variant associated with both traits in or near (+-100kb) the *ASGR1* gene.

Outcome	SNPs	Prior for both traits	H_0	H_1	H_2	H_3	H_4	Conditional H_4
Lifespan	570	1.0E-05	1.3E-73	0.575	1.4E-75	0.006	0.419	0.986
Lifespan	570	1.0E-06	2.0E-73	0.923	2.2E-75	0.009	0.067	0.877
ApoB	916	1.0E-05	9.5E-158	4.3E-85	2.2E-76	4.8E-08	>0.999	>0.999
ApoB	916	1.0E-06	9.5E-157	4.3E-84	2.2E-75	4.8E-07	>0.999	>0.999
TG	916	1.0E-05	4.0E-86	1.8E-13	5.6E-76	0.002	0.998	0.998
TG	916	1.0E-06	4.0E-85	1.8E-12	5.5E-75	0.015	0.985	0.985
ApoA	916	1.0E-05	2.9E-74	0.132	1.4E-75	0.005	0.862	0.994
ApoA	916	1.0E-06	1.3E-73	0.591	6.1E-75	0.024	0.385	0.941
HDL-cholesterol	916	1.0E-05	4.4E-74	0.203	1.5E-74	0.070	0.726	0.912
HDL-cholesterol	916	1.0E-06	1.3E-73	0.587	4.5E-74	0.203	0.210	0.508
Total cholesterol	916	1.0E-05	3.7E-116	1.7E-43	2.2E-76	4.8E-08	>0.999	>0.999
Total cholesterol	916	1.0E-06	3.7E-115	1.7E-42	2.2E-75	4.8E-07	>0.999	>0.999
High cholesterol	916	1.0E-05	1.8E-79	8.2E-07	3.7E-75	0.016	0.984	0.984
High cholesterol	916	1.0E-06	1.6E-78	7.2E-06	3.2E-74	0.139	0.861	0.861
CAD	916	1.0E-05	1.5E-73	0.698	7.4E-76	0.003	0.299	0.990
CAD	916	1.0E-06	2.1E-73	0.955	1.0E-75	0.004	0.041	0.906
ALP	916	1.0E-05	<4.9E-324	<4.9E-324	2.2E-76	4.8E-08	>0.999	>0.999
ALP	916	1.0E-06	<4.9E-324	<4.9E-324	2.2E-75	4.8E-07	>0.999	>0.999
AST	916	1.0E-05	3.1E-83	1.4E-10	2.2E-73	>0.999	1.7E-08	1.7E-08
AST	916	1.0E-06	3.1E-83	1.4E-10	2.2E-73	>0.999	1.7E-09	1.7E-09
GGT	916	1.0E-05	7.9E-77	3.6E-04	3.4E-76	5.5E-04	0.999	0.999
GGT	916	1.0E-06	7.9E-76	0.004	3.3E-75	0.005	0.991	0.995
Albumin	916	1.0E-05	2.5E-96	1.2E-23	2.8E-76	2.9E-04	>0.999	>0.999
Albumin	916	1.0E-06	2.5E-95	1.2E-22	2.8E-75	0.003	0.997	0.997
HbA1c	916	1.0E-05	1.1E-73	0.522	1.8E-74	0.082	0.396	0.829

HbA1c	916	1.0E-06	1.8E-73	0.811	2.8E-74	0.127	0.062	0.326
Haemoglobin	916	1.0E-05	2.3E-84	1.1E-11	5.4E-76	0.001	0.999	0.999
Haemoglobin	916	1.0E-06	2.3E-83	1.0E-10	5.3E-75	0.015	0.985	0.985
Haematocrit	916	1.0E-05	9.2E-81	4.2E-08	8.3E-76	0.003	0.997	0.997
Haematocrit	916	1.0E-06	9.0E-80	4.1E-07	8.1E-75	0.027	0.973	0.973
Red blood cell count	916	1.0E-05	2.1E-75	0.009	8.7E-76	0.003	0.988	0.997
Red blood cell count	916	1.0E-06	1.9E-74	0.085	7.8E-75	0.027	0.888	0.971
Reticulocyte count	916	1.0E-05	1.9E-75	0.009	3.1E-76	4.2E-04	0.991	>0.999
Reticulocyte count	916	1.0E-06	1.7E-74	0.079	2.9E-75	0.004	0.917	0.996
Calcium	916	1.0E-05	7.1E-77	3.3E-04	5.8E-76	0.002	0.998	0.998
Calcium	916	1.0E-06	7.0E-76	0.003	5.7E-75	0.016	0.980	0.983
SHBG	916	1.0E-05	<4.9E-324	<4.9E-324	2.2E-73	>0.999	2.6E-73	2.6E-73
SHBG	916	1.0E-06	<4.9E-324	<4.9E-324	2.2E-73	>0.999	2.6E-74	2.6E-74
IGF-1	916	1.0E-05	2.6E-78	1.2E-05	7.9E-76	0.003	0.997	0.997
IGF-1	916	1.0E-06	2.5E-77	1.1E-04	7.7E-75	0.026	0.974	0.974
CRP	916	1.0E-05	8.8E-86	4.1E-13	2.2E-76	2.1E-05	>0.999	>0.999
CRP	916	1.0E-06	8.8E-85	4.0E-12	2.2E-75	2.1E-04	>0.999	>0.999
Testicular problems	916	1.0E-05	4.9E-74	0.224	3.6E-76	8.8E-04	0.775	0.999
Testicular problems	916	1.0E-06	1.6E-73	0.741	1.2E-75	0.003	0.256	0.989

a. ALP, alkaline phosphatase; ApoA, apolipoprotein A; ApoB, apolipoprotein B; AST, aspartate aminotransferase; CAD, coronary artery disease; CRP, C-reactive protein; GGT, gamma glutamyltransferase; HbA1c, glycated haemoglobin; HDL, high-density lipoprotein; IGF-1, insulin-like growth factor 1; LDL, low-density lipoprotein; SHBG, sex hormone-binding globulin; TG, triglycerides.

b. H_0, no association with either trait; H_1, association with LDL-cholesterol only; H_2, association with the outcome only; H_3, associations of two independent variants and one for each trait; H_4, associations of one shared variant with both traits; conditional H_4, associations of one shared variant with both traits conditional on the presence of a variant associated with the outcome ($H_4/(H_2+H_3+H_4)$).

Supplemental Table S6. Mendelian randomization inverse variance weighted estimates for genetically mimicked ASGR1 inhibitors (instrumented by rs55714927 and rs150688657) on significant outcomes.

Sex	Outcome	Beta	95% CI	*P* value
Overall	ApoB	-0.94	-1.09, -0.79	1.1E-33
Women	ApoB	-0.95	-1.16, -0.74	1.7E-19
Men	ApoB	-0.93	-1.16, -0.71	5.8E-16
Overall	TG	-0.46	-0.61, -0.31	1.5E-09
Women	TG	-0.36	-0.57, -0.16	5.6E-04
Men	TG	-0.59	-0.82, -0.36	3.5E-07
Overall	ApoA	-0.24	-0.39, -0.10	1.3E-03
Women	ApoA	-0.19	-0.41, 0.03	9.7E-02
Men	ApoA	-0.35	-0.59, -0.12	3.4E-03
Overall	HDL-cholesterol	0.00	-0.15, 0.14	9.6E-01
Women	HDL-cholesterol	0.11	-0.11, 0.33	3.1E-01
Men	HDL-cholesterol	-0.13	-0.37, 0.10	2.6E-01
Overall	Total cholesterol	-0.77	-0.92, -0.62	4.0E-24
Women	Total cholesterol	-0.78	-0.99, -0.58	5.2E-14
Men	Total cholesterol	-0.78	-1.00, -0.55	1.1E-11
Overall	High cholesterol	-1.26	-1.71, -0.80	7.6E-08
Women	High cholesterol	-1.07	-1.76, -0.37	2.6E-03
Men	High cholesterol	-1.42	-2.04, -0.80	6.4E-06
Overall	CAD	-0.48	-1.11, 0.16	1.4E-01
Women	CAD	-0.32	-1.52, 0.89	6.1E-01
Men	CAD	-0.56	-1.32, 0.21	1.6E-01
Overall	ALP	2.60	2.45, 2.75	8.4E-257
Women	ALP	2.24	2.05, 2.44	2.9E-108
Men	ALP	3.09	2.86, 3.32	1.1E-157
Overall	AST	0.06	-0.08, 0.21	4.0E-01
Women	AST	0.09	-0.11, 0.30	3.7E-01
Men	AST	0.04	-0.19, 0.26	7.6E-01

Overall	GGT	0.19	0.05, 0.34	7.4E-03
Women	GGT	0.23	0.02, 0.43	3.1E-02
Men	GGT	0.20	-0.02, 0.43	7.9E-02
Overall	Albumin	-0.56	-0.72, -0.41	2.7E-12
Women	Albumin	-0.58	-0.80, -0.36	2.4E-07
Men	Albumin	-0.55	-0.78, -0.32	2.6E-06
Overall	HbA1c	-0.52	-0.67, -0.37	5.5E-12
Women	HbA1c	-0.65	-0.85, -0.45	1.2E-10
Men	HbA1c	-0.38	-0.60, -0.16	7.6E-04
Overall	Haemoglobin	0.17	0.04, 0.29	7.5E-03
Women	Haemoglobin	0.12	-0.09, 0.32	2.7E-01
Men	Haemoglobin	0.33	0.10, 0.55	4.2E-03
Overall	Haematocrit	0.13	0.00, 0.25	4.3E-02
Women	Haematocrit	0.09	-0.12, 0.30	4.0E-01
Men	Haematocrit	0.25	0.02, 0.47	3.2E-02
Overall	Red blood cell count	0.12	-0.01, 0.25	7.0E-02
Women	Red blood cell count	0.06	-0.15, 0.27	5.7E-01
Men	Red blood cell count	0.25	0.03, 0.47	2.7E-02
Overall	Reticulocyte count	0.21	0.05, 0.36	8.3E-03
Women	Reticulocyte count	0.22	0.01, 0.43	4.0E-02
Men	Reticulocyte count	0.20	-0.03, 0.42	9.0E-02
Overall	Calcium	-0.30	-0.46, -0.14	2.5E-04
Women	Calcium	-0.29	-0.50, -0.07	1.0E-02
Men	Calcium	-0.31	-0.55, -0.08	8.7E-03
Overall	SHBG	2.58	2.44, 2.73	2.7E-266
Women	SHBG	2.63	2.41, 2.85	1.3E-119
Men	SHBG	3.17	2.94, 3.40	1.3E-164
Overall	IGF-1	0.45	0.30, 0.60	4.3E-09
Women	IGF-1	0.32	0.12, 0.53	1.8E-03
Men	IGF-1	0.60	0.38, 0.83	1.0E-07

Overall	CRP	0.37	0.22, 0.53	1.7E-06
Women	CRP	0.29	0.09, 0.50	5.7E-03
Men	CRP	0.47	0.25, 0.70	4.1E-05
Men	Testicular problems	-2.08	-6.43, 2.27	3.5E-01

a. ALP, alkaline phosphatase; ApoA, apolipoprotein A; ApoB, apolipoprotein B; AST, aspartate aminotransferase; CAD, coronary artery disease; CI, confidence interval; CRP, C-reactive protein; GGT, gamma glutamyltransferase; HbA1c, glycated haemoglobin; HDL, high-density lipoprotein; IGF-1, insulin-like growth factor 1; LDL, low-density lipoprotein; SHBG, sex hormone-binding globulin; TG, triglycerides.

b. Estimates are expressed in standard deviation (SD) for continuous outcomes, and in log odds for binary outcomes per SD decrease in LDL-cholesterol.

Supplemental Table S7. Mendelian randomization inverse variance weighted estimates for genetically mimicked statins, PCSK9 inhibitors and ezetimibe (instrumented by all relevant SNPs along with their correlations) on significant outcomes identified for ASGR1 inhibitors.

Therapy	Sex	Outcome	Beta	95% CI	*P* value
Statins	Overall	ApoB	-0.74	-0.82, -0.66	1.8E-67
Statins	Women	ApoB	-0.84	-0.94, -0.75	1.5E-65
Statins	Men	ApoB	-0.62	-0.71, -0.53	1.9E-39
Statins	Overall	TG	-0.08	-0.19, 0.02	1.3E-01
Statins	Women	TG	-0.07	-0.18, 0.05	2.5E-01
Statins	Men	TG	-0.10	-0.26, 0.05	1.9E-01
Statins	Overall	ApoA	0.06	-0.00, 0.12	6.7E-02
Statins	Women	ApoA	0.07	-0.02, 0.16	1.4E-01
Statins	Men	ApoA	0.05	-0.04, 0.15	2.9E-01
Statins	Overall	HDL-cholesterol	-0.10	-0.17, -0.03	7.1E-03
Statins	Women	HDL-cholesterol	-0.09	-0.18, -0.00	4.7E-02
Statins	Men	HDL-cholesterol	-0.12	-0.22, -0.03	1.3E-02
Statins	Overall	Total cholesterol	-0.78	-0.88, -0.68	1.2E-52
Statins	Women	Total cholesterol	-0.89	-1.01, -0.77	7.4E-48
Statins	Men	Total cholesterol	-0.68	-0.78, -0.59	1.7E-45
Statins	Overall	High cholesterol	-1.32	-1.52, -1.13	2.5E-40
Statins	Women	High cholesterol	-1.58	-1.90, -1.26	7.4E-22
Statins	Men	High cholesterol	-1.13	-1.40, -0.86	1.8E-16
Statins	Overall	CAD	-0.46	-0.79, -0.14	5.4E-03
Statins	Women	CAD	-0.62	-1.16, -0.09	2.3E-02
Statins	Men	CAD	-0.41	-0.83, 0.02	5.9E-02
Statins	Overall	ALP	-0.08	-0.14, -0.02	1.1E-02
Statins	Women	ALP	-0.03	-0.12, 0.05	4.4E-01
Statins	Men	ALP	-0.14	-0.23, -0.05	3.0E-03
Statins	Overall	AST	-0.10	-0.21, 0.02	1.1E-01
Statins	Women	AST	-0.09	-0.20, 0.02	1.0E-01

Statins	Men	AST	-0.11	-0.26, 0.04	1.5E-01
Statins	Overall	GGT	-0.01	-0.11, 0.10	9.2E-01
Statins	Women	GGT	0.02	-0.16, 0.20	8.3E-01
Statins	Men	GGT	-0.04	-0.13, 0.05	3.9E-01
Statins	Overall	Albumin	-0.04	-0.11, 0.02	2.1E-01
Statins	Women	Albumin	-0.07	-0.16, 0.02	1.2E-01
Statins	Men	Albumin	-0.01	-0.10, 0.09	9.2E-01
Statins	Overall	HbA1c	0.16	0.09, 0.22	2.0E-06
Statins	Women	HbA1c	0.16	0.06, 0.26	2.2E-03
Statins	Men	HbA1c	0.15	0.06, 0.24	1.2E-03
Statins	Overall	Haemoglobin	-0.06	-0.12, -0.00	4.9E-02
Statins	Women	Haemoglobin	-0.07	-0.16, 0.02	1.1E-01
Statins	Men	Haemoglobin	-0.08	-0.18, 0.02	1.1E-01
Statins	Overall	Haematocrit	-0.03	-0.10, 0.04	4.4E-01
Statins	Women	Haematocrit	-0.05	-0.14, 0.04	3.1E-01
Statins	Men	Haematocrit	-0.01	-0.11, 0.08	7.9E-01
Statins	Overall	Red blood cell count	-0.05	-0.10, 0.01	7.6E-02
Statins	Women	Red blood cell count	-0.08	-0.18, 0.03	1.5E-01
Statins	Men	Red blood cell count	-0.04	-0.13, 0.05	4.3E-01
Statins	Overall	Reticulocyte count	0.05	-0.03, 0.12	2.2E-01
Statins	Women	Reticulocyte count	0.05	-0.06, 0.15	3.8E-01
Statins	Men	Reticulocyte count	0.05	-0.04, 0.14	2.9E-01
Statins	Overall	Calcium	-0.18	-0.25, -0.12	4.9E-08
Statins	Women	Calcium	-0.25	-0.34, -0.16	3.7E-08
Statins	Men	Calcium	-0.10	-0.19, -0.00	4.3E-02
Statins	Overall	SHBG	-0.13	-0.19, -0.07	1.4E-05
Statins	Women	SHBG	-0.22	-0.32, -0.11	7.8E-05
Statins	Men	SHBG	-0.06	-0.15, 0.03	2.1E-01
Statins	Overall	IGF-1	-0.08	-0.14, -0.02	1.4E-02
Statins	Women	IGF-1	-0.04	-0.13, 0.04	2.9E-01

Statins	Men	IGF-1	-0.12	-0.21, -0.03	1.1E-02
Statins	Overall	CRP	-0.08	-0.16, 0.00	6.2E-02
Statins	Women	CRP	-0.03	-0.17, 0.12	7.0E-01
Statins	Men	CRP	-0.13	-0.23, -0.04	4.3E-03
Statins	Men	Testicular problems	-0.35	-1.99, 1.28	6.7E-01
PCSK9 inhibitors	Overall	ApoB	-0.73	-0.83, -0.63	1.0E-46
PCSK9 inhibitors	Women	ApoB	-0.75	-0.83, -0.67	3.0E-72
PCSK9 inhibitors	Men	ApoB	-0.70	-0.85, -0.56	1.3E-21
PCSK9 inhibitors	Overall	TG	0.02	-0.07, 0.11	5.9E-01
PCSK9 inhibitors	Women	TG	-0.03	-0.15, 0.08	5.8E-01
PCSK9 inhibitors	Men	TG	0.09	-0.04, 0.22	1.7E-01
PCSK9 inhibitors	Overall	ApoA	0.12	0.06, 0.18	4.9E-05
PCSK9 inhibitors	Women	ApoA	0.12	0.03, 0.20	8.9E-03
PCSK9 inhibitors	Men	ApoA	0.15	0.05, 0.24	3.4E-03
PCSK9 inhibitors	Overall	HDL-cholesterol	-0.01	-0.07, 0.05	6.8E-01
PCSK9 inhibitors	Women	HDL-cholesterol	0.01	-0.08, 0.10	8.1E-01
PCSK9 inhibitors	Men	HDL-cholesterol	-0.04	-0.15, 0.07	4.4E-01
PCSK9 inhibitors	Overall	Total cholesterol	-0.65	-0.74, -0.56	2.4E-49
PCSK9 inhibitors	Women	Total cholesterol	-0.68	-0.76, -0.60	8.7E-63
PCSK9 inhibitors	Men	Total cholesterol	-0.64	-0.77, -0.51	8.7E-22
PCSK9 inhibitors	Overall	High cholesterol	-1.15	-1.34, -0.96	2.1E-32
PCSK9 inhibitors	Women	High cholesterol	-1.26	-1.53, -0.99	6.5E-20
PCSK9 inhibitors	Men	High cholesterol	-1.07	-1.31, -0.83	1.6E-18
PCSK9 inhibitors	Overall	CAD	-0.52	-0.82, -0.23	5.3E-04
PCSK9 inhibitors	Women	CAD	-0.36	-0.83, 0.11	1.4E-01
PCSK9 inhibitors	Men	CAD	-0.61	-0.96, -0.25	8.9E-04
PCSK9 inhibitors	Overall	ALP	0.07	0.01, 0.12	2.6E-02
PCSK9 inhibitors	Women	ALP	0.08	-0.00, 0.15	5.5E-02
PCSK9 inhibitors	Men	ALP	0.05	-0.04, 0.15	2.7E-01
PCSK9 inhibitors	Overall	AST	0.06	-0.01, 0.13	9.7E-02

PCSK9 inhibitors	Women	AST	0.06	-0.05, 0.17	3.0E-01
PCSK9 inhibitors	Men	AST	0.06	-0.03, 0.16	2.0E-01
PCSK9 inhibitors	Overall	GGT	0.09	0.04, 0.15	1.0E-03
PCSK9 inhibitors	Women	GGT	0.09	0.01, 0.17	2.4E-02
PCSK9 inhibitors	Men	GGT	0.11	-0.01, 0.23	8.1E-02
PCSK9 inhibitors	Overall	Albumin	0.04	-0.06, 0.13	4.5E-01
PCSK9 inhibitors	Women	Albumin	0.06	-0.04, 0.15	2.3E-01
PCSK9 inhibitors	Men	Albumin	0.01	-0.09, 0.12	8.1E-01
PCSK9 inhibitors	Overall	HbA1c	-0.02	-0.07, 0.04	5.8E-01
PCSK9 inhibitors	Women	HbA1c	-0.04	-0.11, 0.04	3.8E-01
PCSK9 inhibitors	Men	HbA1c	0.00	-0.08, 0.09	9.4E-01
PCSK9 inhibitors	Overall	Haemoglobin	0.11	0.06, 0.16	6.8E-06
PCSK9 inhibitors	Women	Haemoglobin	0.08	-0.00, 0.16	5.7E-02
PCSK9 inhibitors	Men	Haemoglobin	0.21	0.12, 0.30	5.2E-06
PCSK9 inhibitors	Overall	Haematocrit	0.09	0.04, 0.14	2.1E-04
PCSK9 inhibitors	Women	Haematocrit	0.06	-0.02, 0.14	1.6E-01
PCSK9 inhibitors	Men	Haematocrit	0.18	0.09, 0.27	8.2E-05
PCSK9 inhibitors	Overall	Red blood cell count	0.09	0.04, 0.14	9.5E-04
PCSK9 inhibitors	Women	Red blood cell count	0.05	-0.03, 0.13	2.4E-01
PCSK9 inhibitors	Men	Red blood cell count	0.16	0.05, 0.27	6.2E-03
PCSK9 inhibitors	Overall	Reticulocyte count	0.14	0.08, 0.20	4.9E-06
PCSK9 inhibitors	Women	Reticulocyte count	0.14	0.05, 0.22	1.4E-03
PCSK9 inhibitors	Men	Reticulocyte count	0.15	0.05, 0.25	3.7E-03
PCSK9 inhibitors	Overall	Calcium	-0.02	-0.09, 0.05	6.3E-01
PCSK9 inhibitors	Women	Calcium	-0.03	-0.12, 0.05	4.5E-01
PCSK9 inhibitors	Men	Calcium	0.00	-0.12, 0.11	9.9E-01
PCSK9 inhibitors	Overall	SHBG	0.06	0.01, 0.12	3.1E-02
PCSK9 inhibitors	Women	SHBG	0.06	-0.02, 0.15	1.6E-01
PCSK9 inhibitors	Men	SHBG	0.08	-0.06, 0.21	2.6E-01
PCSK9 inhibitors	Overall	IGF-1	-0.01	-0.08, 0.06	7.5E-01

PCSK9 inhibitors	Women	IGF-1	0.02	-0.06, 0.10	7.0E-01
PCSK9 inhibitors	Men	IGF-1	-0.04	-0.14, 0.05	3.6E-01
PCSK9 inhibitors	Overall	CRP	0.08	0.00, 0.16	4.9E-02
PCSK9 inhibitors	Women	CRP	0.10	-0.00, 0.20	5.9E-02
PCSK9 inhibitors	Men	CRP	0.06	-0.03, 0.15	2.3E-01
PCSK9 inhibitors	Men	Testicular problems	-0.40	-2.00, 1.21	6.3E-01
Ezetimibe	Overall	ApoB	-0.67	-0.81, -0.54	5.6E-23
Ezetimibe	Women	ApoB	-0.81	-0.97, -0.64	5.0E-22
Ezetimibe	Men	ApoB	-0.53	-0.74, -0.31	2.6E-06
Ezetimibe	Overall	TG	-0.14	-0.33, 0.05	1.5E-01
Ezetimibe	Women	TG	-0.15	-0.50, 0.20	4.0E-01
Ezetimibe	Men	TG	-0.13	-0.40, 0.13	3.2E-01
Ezetimibe	Overall	ApoA	0.16	0.04, 0.28	7.5E-03
Ezetimibe	Women	ApoA	0.09	-0.09, 0.28	3.3E-01
Ezetimibe	Men	ApoA	0.27	0.08, 0.46	4.9E-03
Ezetimibe	Overall	HDL-cholesterol	0.13	-0.00, 0.25	5.0E-02
Ezetimibe	Women	HDL-cholesterol	0.09	-0.16, 0.33	4.9E-01
Ezetimibe	Men	HDL-cholesterol	0.20	0.01, 0.39	3.5E-02
Ezetimibe	Overall	Total cholesterol	-0.69	-0.80, -0.57	5.6E-30
Ezetimibe	Women	Total cholesterol	-0.82	-0.98, -0.66	4.4E-23
Ezetimibe	Men	Total cholesterol	-0.56	-0.75, -0.36	1.6E-08
Ezetimibe	Overall	High cholesterol	-1.55	-1.91, -1.20	4.9E-18
Ezetimibe	Women	High cholesterol	-1.55	-2.09, -1.02	1.2E-08
Ezetimibe	Men	High cholesterol	-1.58	-2.05, -1.10	6.7E-11
Ezetimibe	Overall	CAD	-0.85	-1.70, 0.00	5.1E-02
Ezetimibe	Women	CAD	-0.88	-2.18, 0.42	1.9E-01
Ezetimibe	Men	CAD	-0.87	-1.87, 0.13	8.8E-02
Ezetimibe	Overall	ALP	-0.16	-0.31, -0.01	3.8E-02
Ezetimibe	Women	ALP	-0.19	-0.35, -0.04	1.6E-02
Ezetimibe	Men	ALP	-0.11	-0.35, 0.13	3.7E-01

Ezetimibe	Overall	AST	-0.04	-0.20, 0.12	6.2E-01
Ezetimibe	Women	AST	-0.01	-0.17, 0.16	9.3E-01
Ezetimibe	Men	AST	-0.08	-0.26, 0.10	3.6E-01
Ezetimibe	Overall	GGT	-0.16	-0.36, 0.04	1.3E-01
Ezetimibe	Women	GGT	-0.26	-0.46, -0.05	1.4E-02
Ezetimibe	Men	GGT	-0.06	-0.31, 0.19	6.4E-01
Ezetimibe	Overall	Albumin	-0.06	-0.24, 0.12	5.3E-01
Ezetimibe	Women	Albumin	-0.05	-0.22, 0.13	6.1E-01
Ezetimibe	Men	Albumin	-0.07	-0.37, 0.23	6.3E-01
Ezetimibe	Overall	HbA1c	0.35	0.13, 0.58	2.3E-03
Ezetimibe	Women	HbA1c	0.36	0.08, 0.64	1.1E-02
Ezetimibe	Men	HbA1c	0.34	0.05, 0.63	2.0E-02
Ezetimibe	Overall	Haemoglobin	0.00	-0.11, 0.11	9.7E-01
Ezetimibe	Women	Haemoglobin	-0.10	-0.26, 0.07	2.5E-01
Ezetimibe	Men	Haemoglobin	0.10	-0.19, 0.39	5.1E-01
Ezetimibe	Overall	Haematocrit	0.05	-0.05, 0.15	3.7E-01
Ezetimibe	Women	Haematocrit	-0.02	-0.19, 0.14	7.9E-01
Ezetimibe	Men	Haematocrit	0.15	-0.14, 0.45	3.1E-01
Ezetimibe	Overall	Red blood cell count	0.07	-0.08, 0.23	3.4E-01
Ezetimibe	Women	Red blood cell count	0.05	-0.16, 0.26	6.3E-01
Ezetimibe	Men	Red blood cell count	0.13	-0.10, 0.36	2.6E-01
Ezetimibe	Overall	Reticulocyte count	0.12	-0.10, 0.34	2.9E-01
Ezetimibe	Women	Reticulocyte count	0.14	-0.12, 0.40	3.0E-01
Ezetimibe	Men	Reticulocyte count	0.09	-0.13, 0.32	4.1E-01
Ezetimibe	Overall	Calcium	-0.05	-0.28, 0.17	6.3E-01
Ezetimibe	Women	Calcium	-0.15	-0.37, 0.07	1.8E-01
Ezetimibe	Men	Calcium	0.06	-0.19, 0.31	6.3E-01
Ezetimibe	Overall	SHBG	-0.07	-0.30, 0.15	5.2E-01
Ezetimibe	Women	SHBG	-0.08	-0.30, 0.14	4.9E-01
Ezetimibe	Men	SHBG	-0.09	-0.41, 0.23	5.9E-01

Ezetimibe	Overall	IGF-1	0.15	-0.06, 0.37	1.6E-01
Ezetimibe	Women	IGF-1	0.18	-0.04, 0.41	1.1E-01
Ezetimibe	Men	IGF-1	0.12	-0.13, 0.36	3.6E-01
Ezetimibe	Overall	CRP	-0.04	-0.35, 0.26	7.7E-01
Ezetimibe	Women	CRP	-0.09	-0.57, 0.38	7.0E-01
Ezetimibe	Men	CRP	0.01	-0.16, 0.19	8.7E-01
Ezetimibe	Men	Testicular problems	3.17	-2.35, 8.68	2.6E-01

a. ALP, alkaline phosphatase; ApoA, apolipoprotein A; ApoB, apolipoprotein B; AST, aspartate aminotransferase; CAD, coronary artery disease; CI, confidence interval; CRP, C-reactive protein; GGT, gamma glutamyltransferase; HbA1c, glycated haemoglobin; HDL, high-density lipoprotein; IGF-1, insulin-like growth factor 1; LDL, low-density lipoprotein; SHBG, sex hormone-binding globulin; TG, triglycerides.

b. Estimates are expressed in standard deviation (SD) for continuous outcomes, and in log odds for binary outcomes per SD decrease in LDL-cholesterol.

Supplemental Table S8. Mendelian randomization inverse variance weighted estimates for genetically mimicked ASGR1 inhibitors on significant outcomes in replication studies.

Outcome	Consortium	PMID/Source	Overlap with UK Biobank	Population	Number (cases/controls)	SNPs	Beta	95% CI	*P* value
ApoB	Meta-analysis	27005778	No	European	20690	2	-0.68	-1.37 to 0.02	5.6E-02
TG	GLGC	34887591	No	European	864240	1	-0.26	-0.37 to -0.16	3.0E-07
ApoA1	Meta-analysis	27005778	No	European	20687	2	-0.23	-0.94 to 0.49	5.3E-01
HDL-cholesterol	GLGC	34887591	No	European	888227	1	0.25	0.15 to 0.35	8.5E-07
Total cholesterol	GLGC	34887591	No	European	930672	1	-0.88	-0.98 to -0.78	1.1E-70
CAD	CARDIoGRAMplusC4D	26343387	No	Mixed (mainly European)	60801/123504	2	-0.93	-1.65 to -0.22	1.0E-02
ALP	Biobank Japan	34594039	No	East Asian	118886	1	2.94	2.62 to 3.25	6.3E-74
AST	Biobank Japan	34594039	No	East Asian	150068	1	0.60	0.31 to 0.88	3.9E-05
GGT	Biobank Japan	34594039	No	East Asian	133471	1	0.56	0.28 to 0.85	1.0E-04
Albumin	Biobank Japan	34594039	No	East Asian	120539	1	-0.26	-0.57 to 0.04	9.3E-02
HbA1c	MAGIC	34059833	No	European	146806	1	-0.002	-0.16 to 0.16	9.8E-01
Haemoglobin	Meta-analysis	32888493	Yes	European	563946	1	0.35	0.25 to 0.45	7.2E-12
Haematocrit	Meta-analysis	32888493	Yes	European	562259	1	0.36	0.26 to 0.46	4.9E-12
Red blood cell count	Meta-analysis	32888493	Yes	European	545203	1	0.24	0.14 to 0.34	2.3E-06

Reticulocyte count	UK Biobank+UK BiLEVE+INTERVAL	27863252	Yes	European	170641	1	0.17	-0.02 to 0.35	7.4E-02
Calcium	Biobank Japan	34594039	No	East Asian	83980	1	0.22	-0.17 to 0.60	2.7E-01
CRP	UK Biobank+CHARGE	35459240	Yes	European	575531	2	0.35	0.22 to 0.49	4.1E-07

a. ALP, alkaline phosphatase; ApoA, apolipoprotein A; ApoB, apolipoprotein B; AST, aspartate aminotransferase; CAD, coronary artery disease; CARDIoGRAMplusC4D, Coronary ARtery DIsease Genome wide Replication and Meta-analysis plus The Coronary Artery Disease Genetics; CHARGE, Cohorts for Heart and Aging Research in Genomic Epidemiology consortia; CI, confidence interval; CRP, C-reactive protein; GGT, gamma glutamyltransferase; GLGC, Global Lipids Genetics Consortium; HbA1c, glycated haemoglobin; HDL, high-density lipoprotein; MAGIC, the Meta-Analyses of Glucose and Insulin-related traits Consortium; OR, odds ratio; SD, standard deviation; TG, triglycerides; UK BiLEVE, UK Biobank Lung Exome Variant Evaluation.

b. One SNP for ASGR1 inhibitors was rs186021206, and two independent SNPs were rs55714927 and rs150688657 in people of European ancestry; one SNP for ASGR1 inhibitors was rs55714927 in East Asians.

c. Estimates are expressed in percentage for HbA1c, in SD for other continuous outcomes, and in log odds for binary outcomes per SD decrease in LDL-cholesterol.

Supplemental Note: R code for Mendelian randomization analysis, phenome-wide association analysis and colocalization analysis.

```
####Mendelian randomization analysis####

library(TwoSampleMR)

library(MendelianRandomization)


#Harmonise exposure data and outcome data#

exposure<-format_data(data_exp,type="exposure",snp_col = 'rsid',beta_col = "beta",
                      se_col = "se",effect_allele_col = "alt",other_allele_col = "ref",
                      eaf_col = "AF",pval_col = "pval")

outcome<-format_data(data_out,type="outcome",snp_col = 'rsid',beta_col = "beta",
                     se_col = "se",effect_allele_col = "alt",other_allele_col = "ref",
                     eaf_col = "AF",pval_col = "pval")

data<-harmonise_data(exposure,outcome,action=2)

data2<-data[data$mr_keep%in% TRUE,]


#Inverse variance weighted estimates for independent genetic variants#

mrinput<-dat_to_MRInput(data2)

MendelianRandomization::mr_ivw(mrinput[[1]])


#Inverse variance weighted estimates for correlated genetic variants along with correlations#

mrinput<-dat_to_MRInput(data2,get_correlations = TRUE)

MendelianRandomization::mr_ivw(mrinput[[1]],correl = TRUE)


####Phenome-wide association analysis####
```

```
#Extract genetic associations from the UK Biobank (Neale lab)#

get_associations <- function(data, rows, snp) {

  for(i in rows) {

    # Create temp directory and temp file

    temp_dir <- tempdir(check = TRUE)

    temp_bgz <- tempfile(fileext = ".bgz")



    # Download data file for row i

    download(url = data$url[i], destfile = temp_bgz, mode = "wb")



    # Extract data for the SNP of interest

    temp_data1 <- read_tsv(temp_bgz,col_select = c("variant","beta","se",'pval'))

    temp_data2<-temp_data1[temp_data1$variant %in% snp,]

    temp_data<-transform(temp_data2,x=i,phenotype_code=data$phenotype_code[i])



    # Export the data to CSV file

    write_excel_csv(temp_data, str_c("extract_phenotypes_", deparse(substitute(data)), ".csv"), append = TRUE)



    # Delete the temp file and directory

    unlink(temp_bgz)

    unlink(temp_dir, recursive = TRUE)



    # Garbage collection to clear memory at the end of the loop
```

```
    gc()
  }
}

####Colocalization analysis####
library(coloc)

coloc_exp<-data_exp[,c("snp","position","beta","varbeta")]
coloc_out<-data_out[,c("snp","position","beta","varbeta")]

#Format data for quantitative traits#
coloc_exp<-as.list(coloc_exp)
coloc_exp$type<-"quant"
coloc_exp$sdY<-sdY #sdY is the population standard deviation of the trait#
check_dataset(coloc_exp)

#Format data for binary traits#
coloc_out <-as.list(coloc_out)
coloc_out $type<-"cc"
coloc_out $s<-s #s is the proportion of cases in the sample#
check_dataset(coloc_out)

#Colocalization analyses using prior probabilities 1.0e-4 for a variant associated with the exposure, 1.0e-4 for a variant associated with the outcome, and 1.0e-5 for a variant associated with both traits#
```

```
coloc.abf(coloc_exp, coloc_out, MAF = NULL, p1 = 1e-04, p2 = 1e-04, p12 = 1e-5)
```

#Colocalization analyses using prior probabilities 1.0e-4 for a variant associated with the exposure, 1.0e-4 for a variant associated with the outcome, and 1.0e-6 for a variant associated with both traits#

```
coloc.abf(coloc_exp, coloc_out, MAF = NULL, p1 = 1e-04, p2 = 1e-04, p12 = 1e-6)
```
